# New genome assembly of the barn owl (*Tyto alba alba*)

**DOI:** 10.1002/ece3.5991

**Published:** 2020-02-19

**Authors:** Anne‐Lyse Ducrest, Samuel Neuenschwander, Emanuel Schmid‐Siegert, Marco Pagni, Clément Train, David Dylus, Yannis Nevers, Alex Warwick Vesztrocy, Luis M. San‐Jose, Mélanie Dupasquier, Christophe Dessimoz, Ioannis Xenarios, Alexandre Roulin, Jérôme Goudet

**Affiliations:** ^1^ Department of Ecology and Evolution University of Lausanne Lausanne Switzerland; ^2^ Vital‐IT Swiss Institute of Bioinformatics Lausanne Switzerland; ^3^ Department of Computational Biology University of Lausanne Lausanne Switzerland; ^4^ Center for Integrative Genomics University of Lausanne Lausanne Switzerland; ^5^ Swiss Institute of Bioinformatics Lausanne Switzerland; ^6^ Center for Life's Origins and Evolution Department of Genetics, Evolution and Environment University College London London UK; ^7^ Laboratory Evolution and Biological Diversity UMR 5174 CNRS University of Toulouse III Paul Sabatier Toulouse France; ^8^ Lausanne Genomic Technologies Facility Lausanne Switzerland

**Keywords:** assembly, barn owl, bird, genome, Strigiformes, *Tytonidae*

## Abstract

New genomic tools open doors to study ecology, evolution, and population genomics of wild animals. For the Barn owl species complex, a cosmopolitan nocturnal raptor, a very fragmented draft genome was assembled for the American species (*Tyto furcata pratincola*) (Jarvis et al. 2014). To improve the genome, we assembled de novo Illumina and Pacific Biosciences (PacBio) long reads sequences of its European counterpart (*Tyto alba alba*). This genome assembly of 1.219 Gbp comprises 21,509 scaffolds and results in a N50 of 4,615,526 bp. BUSCO (Universal Single‐Copy Orthologs) analysis revealed an assembly completeness of 94.8% with only 1.8% of the genes missing out of 4,915 avian orthologs searched, a proportion similar to that found in the genomes of the zebra finch (*Taeniopygia guttata*) or the collared flycatcher (*Ficedula albicollis*). By mapping the reads of the female American barn owl to the male European barn owl reads, we detected several structural variants and identified 70 Mbp of the Z chromosome. The barn owl scaffolds were further mapped to the chromosomes of the zebra finch. In addition, the completeness of the European barn owl genome is demonstrated with 94 of 128 proteins missing in the chicken genome retrieved in the European barn owl transcripts. This improved genome will help future barn owl population genomic investigations.

## Graphical abstract

We assemble the genome of the European barn owl (*Tyto alba alba*) of 1.219 Gbp by mixing Illumina (117×) and Pacific Biosciences (PacBio) long reads (12×) technologies. BUSCO (Universal Single‐Copy Orthologs) analysis revealed an assembly completeness of 94.8% with only 1.8% of the genes missing out of 4,915 avian orthologs searched, a proportion similar to that found in the genomes of the zebra finch (*Taeniopygia guttata*) or the collared flycatcher (*Ficedula albicollis*). In addition, we identify the Z chromosome' scaffolds, 73% of proteins thought to be missing in the chicken.

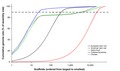

## 1 INTRODUCTION

**Figure 1:**
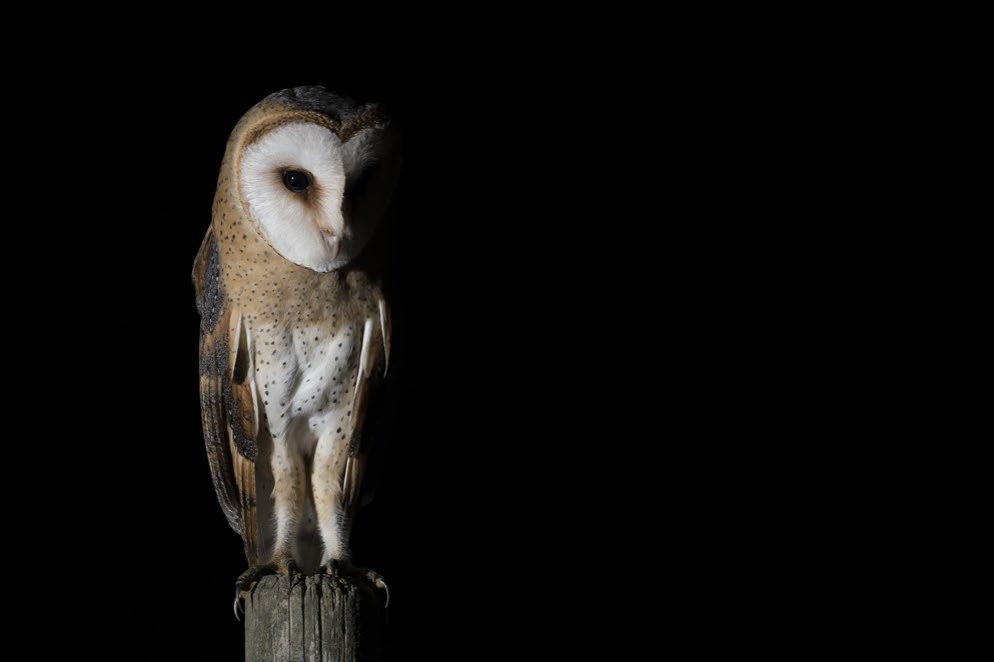
European barn owl (*Tyto alba alba*). ©Guillaume Rapin, Switzerland

The family *Tytonidae* comprises two genera, the bay owls *Phodilus* and the barn owls *Tyto*. Among them the Barn owl species complex (Afro‐European or Western Barn owl *Tyto alba* (Figure 1), the American Barn owl *Tyto furcata* and the Australasian or Eastern Barn owl *Tyto javanica*) have successfully spread all around the world by colonizing all continents except the Antartica (Uva, Packert, Cibois, Fumagalli, & Roulin, 2018). Barn owl adaptation to most ecological conditions (e.g., rain forest, desert, and temperate regions) relies on many notable features such as bill size and plumage color (Romano, Sechaud, Hirzel, & Roulin, 2019a; Romano, Sechaud, & Roulin, 2019b) making this group of bird a relevant biological model. Moreover, as a nocturnal predator, barn owls have developed precise sound localization with asymmetrical ears (Krings, Rosskamp, & Wagner, 2018), performant sensory information processing (Cazettes, Fischer, Beckert, & Pena, 2018; Grothe, 2018; Kraemer, Baxter, Hendrix, & Carr, 2017), silent flight (Wagner, Weger, Klaas, & Schroder, 2017), and a great nocturnal visual acuity (Orlowski, Harmening, & Wagner, 2012; Stemmler et al., 2018). Barn owls also show a great diversity of color patterns both within and between populations which is related to predator‐prey interactions (San‐Jose et al., 2019) and signals aspects of individual quality (Roulin & Ducrest, 2011). Barn owls are thus of high interest for studying a suite of evolutionary ecology questions.

Key toolsets for answering these questions are genomic studies, as they should help identify genes involved in the traits discussed above (e.g., vision, hearing capacity, and color polymorphism), and empower us to perform population genomic analyses and resolve the ancestors of the *Tytonidae* family. The genome of the American barn owl (*T. furcata pratincola,* previously called *Tyto alba pratincola*) had been sequenced by the Beijing Genomics Institute (BGI) (Jarvis et al., 2014). An assembly of 1.12 Gbp was obtained from Illumina short reads with a 27× coverage. This assembly consists of 62,122 scaffolds with a N50 of 52,818 bp (Table 2) and was used to resolve the barn owl's position in the bird tree of life (Jarvis et al., 2014; Prum et al., 2015) and to search for genes associated with low‐light vision (Hanna et al., 2017; Hoglund et al., 2019; Le Duc et al., 2015; Wu et al., 2016). However, the use of draft genome entails problems such as noncontiguous assembly and missing genes, especially in GC‐rich portions of bird genomes (Peona, Weissensteiner, & Suh, 2018). As shown by Warren et al. (2017), adding long reads such as those obtained from single‐molecule real‐time (SMRT, Pacific Biosciences, thereafter called PacBio) improves genome completeness and does not suffer from PCR amplification bias for the sequencing at GC or AT genome‐rich region.

Here, we report a study where we sequenced, assembled, and annotated the genome of a male barn owl (*T. alba alba*) from Switzerland by combining Illumina and PacBio sequencing. We estimated the assembly quality using several metrics and methods, and in particular, we looked for chromosomal synteny with the American barn owl and the zebra finch and for avian “thought lost” genes found in GC‐rich regions. We also examined where the barn owl is positioned in the avian phylogeny by using annotations derived from the American and European barn owls.

## 2 RESULTS

### 2.1 Genome sequencing and assembly

Illumina and PacBio libraries generated a total of 158 Gbp high‐quality sequences (Table 1) and were assembled into 1.219 Gbp. The final assembly contained 21,509 scaffolds of more than 500 bp with a low proportion of undetermined nucleotides (Ns = 0.79%) and a N50 of 4.6 Mbp (Table 2). The heterozygosity was estimated to be 0.373% with kmer plot (Figure S3).

**Table 1:** Metrics of the libraries used for the de novo assembly of the European barn owl

Library type	Length (bp)	Insert size (bp)	Number of reads	Total size (Gbp)	Coverage^a^
Illumina paired‐end^b^	2 × 100	180	243,335,851	48.67	41×
Illumina paired‐end^b^	2 × 100	500	187,046,962	37.41	31×
Illumina paired‐end^b^	2 × 100	500	175,190,557	35.04	29×
Illumina mate‐pair^c^	2 × 100	2,000	38,906,455	7.78	6×
Illumina mate‐pair^c^	2 × 100	5,000	67,900,282	13.58	11×
PacBio^c^	500–49,386		3,169,413	15.03	12×
			in total:	158.00	129×

a The coverage was computed assuming a genome size of 1.219 Gbp equal to the assembly size.

b These libraries were used for assembling and scaffolding.

c These libraries were used solely for scaffolding.

**Table 2:** Comparison of the European barn owl genome assembly metrics and genome completeness using BUSCO to the American barn owl, the zebra finch, the collared flycatcher, and the chicken genome assemblies

	European barn owl	American barn owl	Zebra finch	Collared flycatcher	Chicken
Number of scaffolds	21,509	62,122	37,096	21,428	23,475
Number of scaffolds (≥500 bp)	21,509	57,936	37,096	9,718	23,208
Number of scaffolds (≥1,000 bp)	10,312	47,332	37,094	4,033	22,945
Largest scaffold	22,155,979	502,267	156,412,533	157,563,209	196,202,544
Assembly length	1,219,191,878	1,120,143,088	1,232,135,591	1,118,343,587	1,230,258,557
N50	4,615,526	52,818	62,374,962	64,724,594	82,310,166
N75	1,861,816	25,700	15,652,063	21,727,166	14,109,371
L50	72	5,943	7	6	5
L75	177	13,502	18	13	16
Genome size (Gbp)	1.59	1.59	1.22	1.20	1.25
NG50	2,701,956	29,716	62,374,962	64,724,594	82,310,166
GC (%)	42	40	41	44	43
N's (%)	0.79	0.79	0.75	1.43	0.96
*BUSCO analysis based on 4,915 avian BUSCOs*					
Complete (%)	94.8	84.3	93.6	ND	94.8
Single‐copy (%)	94.0	83.9	90.8	ND	93.8
Duplicated (%)	0.8	0.4	2.8	ND	1.0
Fragmented (%)	3.4	10.6	3.8	ND	2.9
Missing (%)	1.8	5.1	2.6	ND	2.3

Note

All stats were done for scaffolds >500 bp. To evaluate genome completeness, the European barn owl genome was compared with the other genomes using 4,915 conserved avian orthologous genes using BUSCO. Except for genome size (Gbp) all the data are given in bp. The genome size of the barn owl was the mean derived from the C‐values of 1.73 and 1.53 pg of DNA that gave genomic size of 1.69 and 1.50 Gbp with an average of 1.59 Gbp (De Vita et al., 1994; Venturini et al., 1986).

Abbreviation: ND, not done.

**Figure 2:**
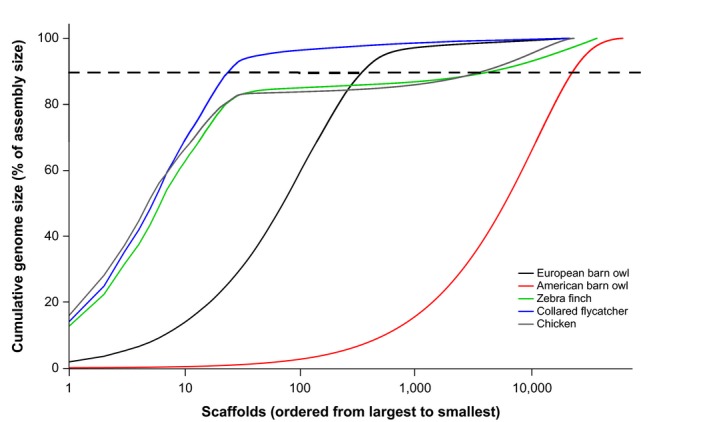
Relation between the number of scaffolds and the percentage of genome assembly of the American barn owl (red), the European barn owl (black), the zebra finch (green), the collared flycatcher (blue), and the chicken (gray). The horizontal dashed lane represents 90% of genome assembly

The assembly metrics of the European barn owl genome are compared with those of the chicken (Gallus_gallus‐5.0), zebra finch (Taeniopygia_gutattata‐3.2.4), collared flycatcher (FicAlb1.5), and American barn owl (ASM68720v1) described in Table S1. The expected maximal assembly size calculated from C‐values ranges from 1.50 to 1.69 Gbp (De Vita, Cavallo, Eleuteri, & Dell'Omo, 1994; Venturini, D'Ambrogi, & Capanna, 1986). The NG50 scaffold length is 2.7 Mbp, a value 23 and 30 times lower than the NG50 of the zebra finch and of the chicken genomes, respectively, but 91 times higher than the NG50 of the American barn owl (Table 2). The longest assembled scaffold (22,155,979 bp) in the European barn owl genome is 7 to 9 times smaller than in the zebra finch, collared flycatcher, and chicken genomes but 44 times larger than in the American barn owl genome (Table 2). The 605 largest scaffolds in the European barn owl cover 95% of its genome assembly, less than the 1,000 scaffolds necessary to cover 95% of the genome assembly of the zebra finch and the chicken, and over 10,000 scaffolds necessary for the American barn owl (Figure 2). The assembly comprises 5.21% of interspersed repetitive elements including SINEs, LINES, LTRs, and unclassified elements; of these the LINES and LTRs were the most abundant with 2.46% and 2.49%, respectively. Noninterspersed repeat elements such as small RNA, satellites, simple repeats, and low complexity represent 1.52% of the assembly. The total percent are 1.4, 1.6, 2.9 times lower than in the zebra finch, flycatcher, and chicken, respectively, and 1.2 times higher than the American barn owl (Table 3).

**Table 3:** Summary of the repetitive elements present in the European barn owl assembly

	Number of elements^a^	Length (bp)	% assembly
*Total interspersed repeats*		*63,491,136*	*5.21*
Total SINEs	2,046	180,446	0.01
ALUs	0	0	0.00
MIRs	0	0	0.00
Total LINEs	73,341	30,014,401	2.46
LINE1	0	0	0.00
LINE2	0	0	0.00
L3/CR1	73,341	30,014,401	2.46
Total LTR elements	6,081	2,932,372	0.24
ERVL	1,553	1,560,499	0.13
ERVL‐MaLRs	0	0	0.00
ERV_classI	1,223	768,947	0.06
ERV_classII	528	340,257	0.03
Total DNA elements	0	0	0.00
hAT‐Charlie	0	0	0.00
TcMar‐Tigger	0	0	0.00
Unclassified	81,182	30,363,917	2.49
*Total noninterspersed repeats*		*18,569,766*	*1.52*
Small RNA	0	0	0.00
Satellites	1	473	0.00
Simple repeats	360,048	15,018,487	1.23
Low complexity	63,493	3,550,806	0.29

a Repeats that contain insertion or deletion were counted as one element.

### 2.2 Quality and completeness assessment

To further assess the quality of the genome of the European barn owl, the Illumina raw reads used to assemble the genome were mapped back to the assembly, resulting in an overall mapping rate above 96%. The completeness of the assembly is supported by searching for Universal Single‐Copy Orthologs (BUSCO) (Simao, Waterhouse, Ioannidis, Kriventseva, & Zdobnov, 2015). 98.2% of the 4,915 avian orthologs are successfully found in the European barn owl assembly, of which 94.8% are complete (94% in single copy and 0.8% duplicated) (Table 2) and 1.8% are missing. The BUSCO analysis demonstrates the European barn owl genome is more complete than the American barn owl, for which only 84.3% of retrieved orthologs are complete and 5.1% are missing (Table 2).

### 2.3 Annotation

The Augustus best model, trained with the chicken Uniprot reference proteome, predicted more than 38,000 proteins, twice as many as the available NCBI American barn owl annotation (Table 4). To further evaluate the annotation completeness, the predicted proteins of the European and American barn owls were compared with a set of 30,252 chicken proteins. Global search (ggsearch) found 10,392 similar proteins in the European barn owl, but only 9,109 in the American barn owl (Table 4). In addition, the predicted protein sets of the European and American barn owl annotations were matched against 978 metazoan orthologues of the Universal Single‐Copy Orthologs implemented in BUSCO (Simao et al., 2015). Though the two annotation sets had the same proportion of reference proteins classified as “complete” (73.9%), the European barn owl annotation set had both fewer missing (13.1% vs. 15.6%) and duplicated (1.1% vs. 3.5%) proteins. The latter is remarkable considering the much larger set of annotated proteins in the European barn owl annotation set.

**Table 4:** Summary metrics and quality assessments of the European barn owl annotations compared with the available American barn owl annotation

	American barn owl	European barn owl
Number of proteins	14,905	38,895
Min length	21	13
Mean length	489	308
Median length	362	471
Max length	22,559	23,122
*Global‐global search of 30,252 supported chicken proteins* a		
Total	9,109	10,392
Unique	8,357	8,946
Duplicates	752	1,446
*BUSCO analysis based on 978 metazoa BUSCOs*		
Complete (%)	73.9	73.9
Single‐copy (%)	70.4	72.8
Duplicated (%)	3.5	1.1
Fragmented (%)	10.4	13.0
Missing (%)	15.6	13.1

Note

The quality assessments were based on the search for chicken and metazoa BUSCO proteins.

a Global‐global search of similar chicken proteins in the European barn owl gene annotations using the chicken (*Gallus gallus* ensembl release 88).

### 2.4 GC content

**Figure 3:**
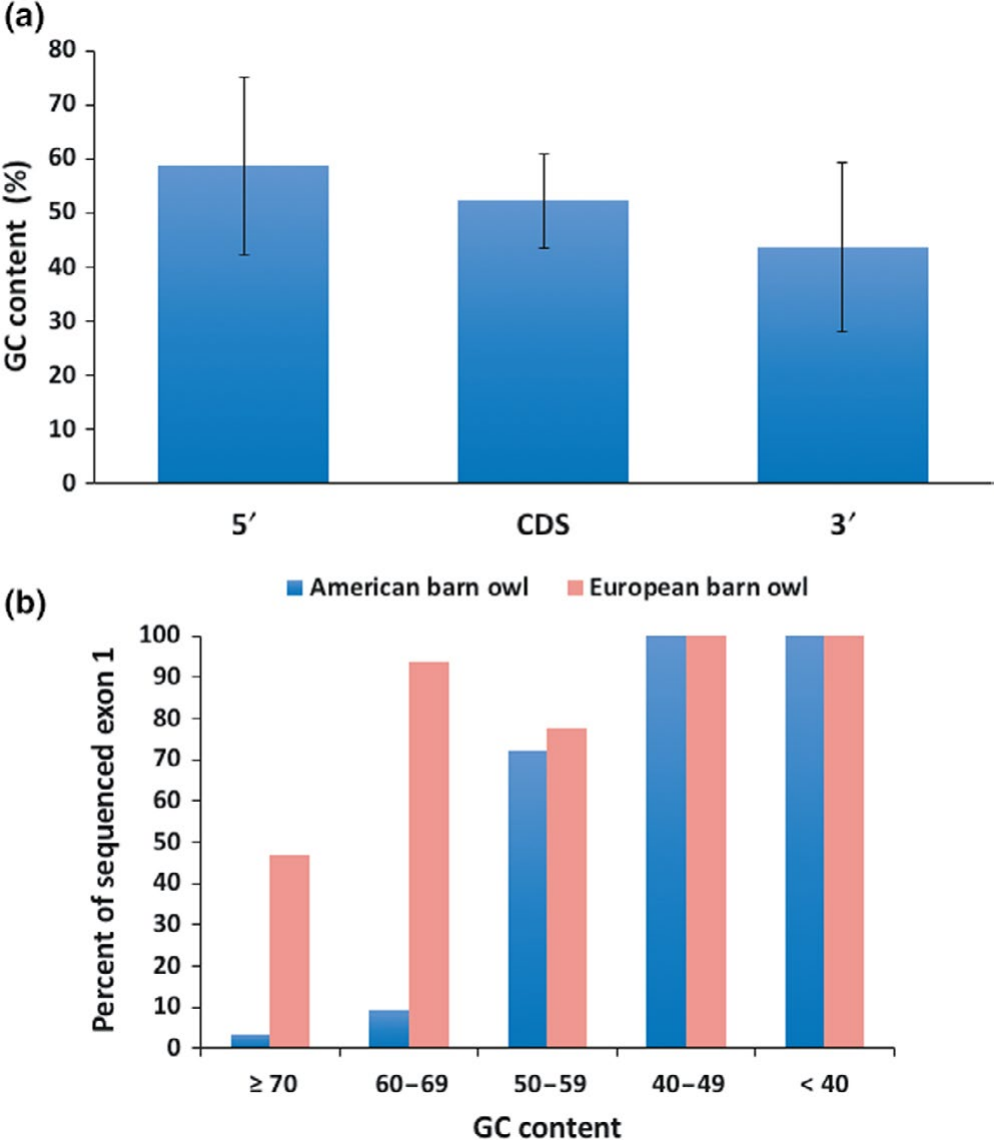
Effect of GC content on genome sequencing of European and American barn owl. (a) GC content of 57 Sanger sequenced genes of the European barn owl. The gene sequences were split in the 5'UTR, the coding sequence (CDS) and the 3'UTR. The mean and the standard deviation for the 57 genes are plotted. (b) Percent of exon 1 that were sequenced by Illumina (American barn owl, blue) or Illumina/PacBio (European barn owl, red) sequencing out of the 57 Sanger sequenced genes, binned according to their GC content. The number of genes for each group of first exon GC content is the following: ≥70:15, 60–69:16, 50–59:9, 40–49:14, <40:3

As shown for primates and birds, distribution of the GC content varies between intra‐ and intergenic regions (Botero‐Castro, Figuet, Tilak, Nabholz, & Galtier, 2017; Qi et al., 2016). In order to investigate the GC content in the European barn owl, we took advantage of 108,132 bp of 57 well‐characterized Sanger sequenced genes (Accession numbers in Table S2). As for humans (Zhang, Kasif, Cantor, & Broude, 2004), the GC content of the genes of the European barn owl varied between and within genes, with 5'UTR being GC‐richer than CDS and 3'UTR (Figure 3a). We observed an average GC content of 51% in exons of the Sanger sequenced genes, which is high compared with the whole genome sequencing value (42%). We also compared the effect of the GC contents on Illumina and PacBio sequencing of the first exon in the same set of genes in the European and American barn owls. Illumina sequencing of high GC‐rich first exon often failed (Figure 3b): Of these first GC‐rich exons (≥70% GC), only 3% of the American barn owl (Illumina only), but 47% of the European barn owl (Illumina and PacBio) were sequenced. This shows that mixing Illumina and PacBio technologies improve the completion of genome sequencing.

### 2.5 Chromosomal synteny of the European barn owl, the American barn owl, and the zebra finch

**Figure 4:**
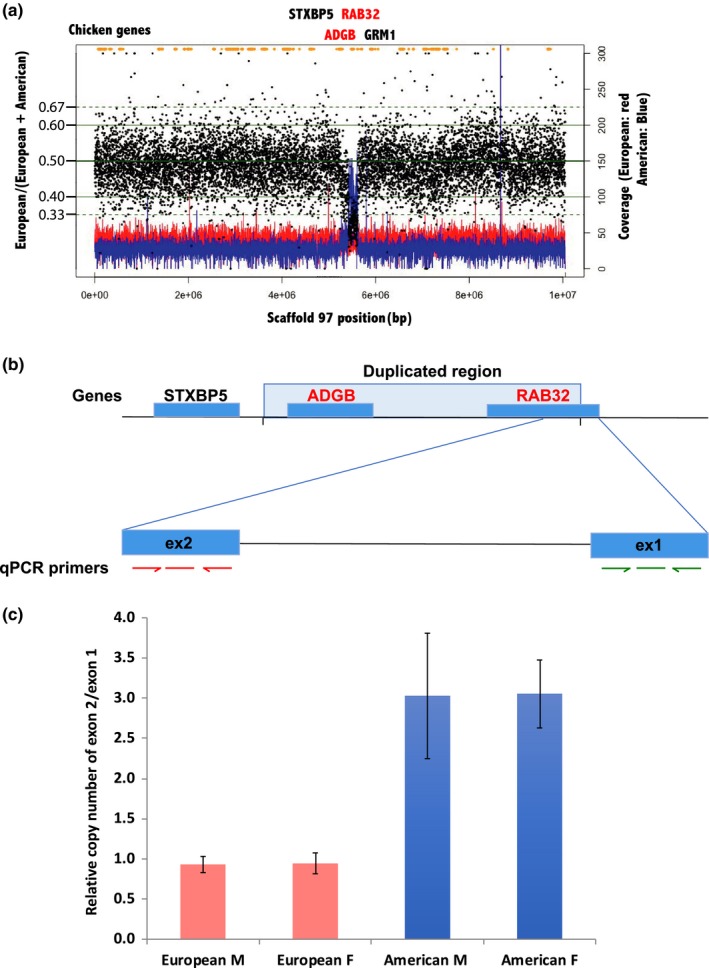
Detection of scaffolds rearrangements in the European and American barn owls. (a) Raw read coverage of the American (blue) and the European (red) barn owls for the scaffold 97 that contains the *RAB32* and androglobin (*ADGB*) genes (written in red), which may be partially duplicated in the American barn owl. The chicken genes surrounding the duplicated region are written in black. The relative coverage of the raw reads of the European barn owl over the sum of the raw reads of the European barn owl plus the American barn owl is depicted with the black dots for each read. A drop of the relative coverage means a duplication in the American barn owl genome. (b) Detection of the duplication in the American barn owl at the Contig 97 by real‐time PCR (qPCR). The copy number of DNA of the exon 2 (in the duplicated region in the American barn owls) and the copy number of the exon 1 (unduplicated) of the RAB32 gene are quantified by qPCR with primers and probes located in the exon 2 and exon 1 of RAB32. (c) Mean value and standard deviation (bars) for the relative copy number of the exon 2 over the exon 1 of *RAB32* in 4 male (M) and 4 female (F) European barn owls and in 3 male and 4 female American barn owls

To distinguish between true rearrangements and technical mis‐assemblies between the two barn owl genomes, we mapped the raw Illumina reads used for the American and European barn owl assemblies to the assembly of the European barn owl (Figure 4a). Similar coverage is observed for most reads between the European and American barn owls, except for few regions. For instance, scaffold 97 showed an increased coverage in the American barn owl compared with the European barn owl (Figure 4a). To verify that the detected variation in the coverage ratio was due to a physical rearrangement, the copy number of genomic DNA in this region was quantified for both barn owl species by real‐time PCR (Figure 4b): We measured relative copy number of the putative duplicated/deleted region to the neighboring genomic region of 8 European and 7 American barn owl individuals. A region spanning 189.3 kbp in the Contig 97 seemed to be triplicated in the American barn owl compared with the European barn owl, since the ratio of the coverage dropped around 0.33 (Figure 4a). Of interest in the concerned region are two genes, the androglobulin (*ADGB*) and the *RAB32* genes. *RAB32* is involved in membrane trafficking in the cells, specially of melanosomes (Stenmark, 2009) and is located in the boundary of the duplication with the first exon being nonduplicated and the second exon in the duplicated region (Figure 4b). Quantitative PCR confirmed a relative increase of 2 to 3 times of the *RAB32* exon 2 over the exon 1 in the seven American barn owls compared with the eight European barn owls (Figure 4c).

**Figure 5:**
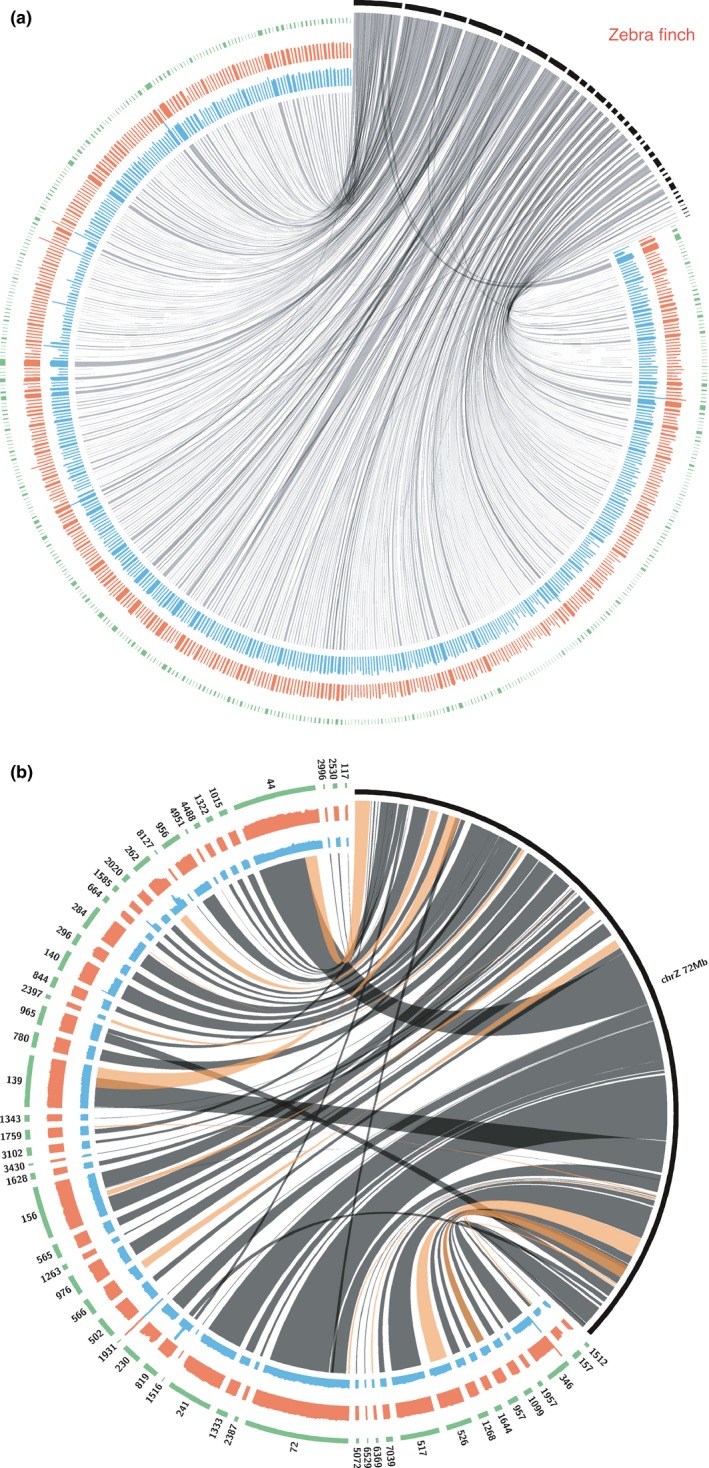
Comparison of European and American barn owl contigs with the zebra finch chromosomes. (a) Chromosomal synteny plot between the zebra finch genome assembled at the chromosome level (black) and the European barn owl scaffolds (green) (a) for all zebra finch chromosomes (black) and (b) for the zebra finch Z chromosome (black). The innermost part represents the localization of the European barn owl scaffolds to the zebra finch chromosomes (gray lines). The outermost line plot represents breadth of coverage of European (red) and American (blue) barn owl scaffolds. For (b) the innermost part represents the localization of the European barn owl scaffolds (gray and orange) to the zebra finch chromosomes with inversions denoted by orange lines

To assess the chromosomal structure of the European barn owl assembly, the scaffolds of the European and American barn owls were aligned to the zebra finch genome and visualized with Circos plots (Krzywinski et al., 2009). The zebra finch genome is one of the closest related species assembled at the chromosomal level (out of 40 haploid zebra finch chromosomes, 2n = 80, 37 have been assembled at the chromosome level, Figure S1; the haploid chromosome number in the barn owl is 46 (Rebholz & Northrop, 1993). A first comparison of all the chromosomes and scaffolds of the Ensembl zebra finch karyotype to the European barn owl scaffolds shows few rearrangements or mis‐assemblies (Figure 5a and Figure S2). These results suggest the overall structure of the European barn owl genome is comparable to the zebra finch genome and confirm the quality of the European barn owl genome assembly.

### 2.6 Identification of contigs belonging to the sexual chromosomes

Since the American barn owl assembly was based on a heterogametic female (ZW) and the European barn owl assembly on a homogametic male (ZZ), comparison of the European and American barn owl genomes could detect the scaffolds belonging to the Z and W chromosomes. Scaffolds belonging to the Z chromosome should be identifiable by twofold reduced coverage of the American reads (female). For the W chromosome, the quality, size, and the low complexity of the DNA sequences notorious for this chromosome hindered its characterization in the American barn owl. 64 scaffolds of the European barn owl, containing 70 Mbp (5.7% of the genome assembly), had a coverage doubled compared with the American barn owl and were assigned to the Z chromosome. When these scaffolds were mapped to the Z chromosome of the zebra finch, the European barn owl scaffolds had a twofold higher coverage compared with American barn owl scaffolds, except for the small scaffold 1931 that appeared to belong to an autosome (Figure 5b). The Z chromosome had the highest number of rearrangements or miss‐assemblies of all chromosomes (Figure 5b and Figure S2).

### 2.7 Avian lost genes

Warren et al. (2017) examined a set of 232 mammalian and lizard proteins that had not been found in any of the 60 bird genomes published so far and another set of 128 mammalian and lizard proteins found in some bird genomes but not in the chicken genome (Tables S3a,b). We investigated whether these two sets of proteins were present in the European barn owl genome. Out of the first set of 232 proteins missing in the bird genomes, 19 are partially found in the European barn owls (Table S3a). From the second set of 128 proteins missing only in the chicken genome, 94 proteins (72.9%) are present in the European barn owl (Table S3b). This again suggests that the European barn owl genome is quite complete by bird standards.

### 2.8 Barn owl position in the tree of life

Previous studies found the position of the barn owl in the Avian phylogeny to be inconsistent when different regions of the genome were considered. In one study (Jarvis et al., 2014), the owl branch was placed either with the raptors (*Accipitridae* and vulture) or with the Coraciimorphae birds, such as mousebirds, cuckoo roller, and trogons using 48 sequenced genomes; in another study, where 122 avian species but only 259 targeted genes (Prum et al., 2015) were used, the barn owl fall within a new clade, the Inopinaves, a sister group of the Coraciimorphae, mousebird, cuckoo roller, trogons, and falcons but not a sister group to the hawks, which form a separated clade, the Eutelluraves. Since genome quality and completeness could impact phylogenetic analyses, we investigated whether the improved European barn owl genome altered the position of this species in the Avian phylogeny. For this, we generated five datasets: (i, ii) two datasets with either the American or the European barn owl as sole owl species; (iii, iv) two datasets with three other owl species in addition to either the American or European barn owl; and (v) one dataset with both American and European barn owls in the same tree.

**Figure 6:**
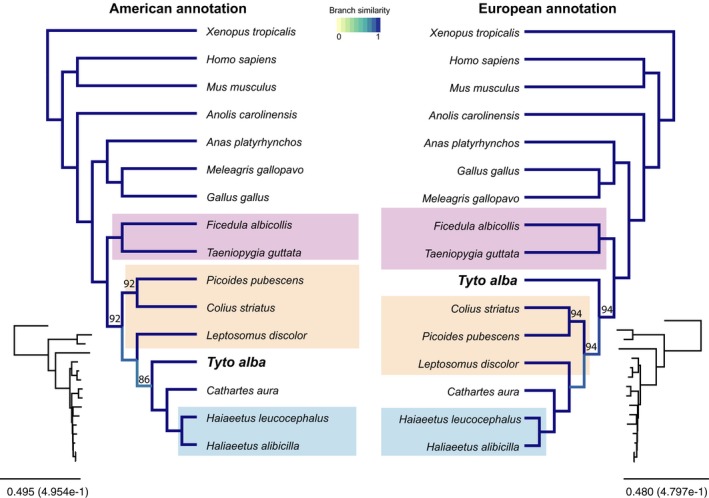
Avian phylogenetic trees based on the American and European barn owl proteins predicted with the American and European barn owl annotations. Depending on the dataset, the position of *Tytonidae* varies on the tree. Left tree used the protein predictions produced by the American annotation, right tree uses the protein predictions produced in this work. Shades of blue show positions in which differences in topology are detected. Nodes without number have a bootstrap support of 100%; Small trees on the sides show the real branch lengths. The purple background represented the group of Passerimorphae, the orange the Coraciimorphae and the light blue the Accipitrimorphae

For the trees with the single owl representative (i, ii), we obtained largely congruent trees, but with some conflicting branches (Figure 6). Support was generally lower with the American barn owl than the European barn owl (86 vs. 94). For the American barn owl annotation, the barn owl is placed outside of the Turkey vulture (*Cathartes aura*) in the tree, consistent with the MP‐Est Tent tree of Figure 3b of Jarvis et al., (2014) and consistent with a separation of Passerimorphae and Coraciimorphae with Accipitrimorphae and the separation of the latter placing barn owl as potential outgroup within the Accipitrimorphae. By contrast, when using the proteome of the European barn owl, this species is positioned as an outgroup to the Coraciimorphae, with Accipitrimorphae clades still being separated from the Passerimorphae. This differs from the proposed position of Prum et al. (2015) where the barn owl is a sister group of the Coraciimorphae (which include the mousebirds, the cuckoo roller, the trogons) and the falcons but not a sister group to the hawks, which form a separate clade.

The inclusion of three additional owl species (iii, iv) resulted in trees that had the same topology as with either American or European barn owl alone, albeit with lower support values for the conflicting branches (Figure S4). Importantly, all owls were grouped together in both trees with a high level of support.

Likewise, when combining the European and American barn owls into a single dataset (v), we obtained a tree grouping the two barn owls with high support, however, splitting *Leptosmus discolor* from the Coraciimorphae clade with low support (Figure S5).

Recent studies have suggested that this may be due to substantial Incomplete Lineage Sorting (ILS) (Houde, Braun, Narula, Minjares, & Mirarab, 2019; Liu et al., 2018; Suh, Smeds, & Ellegren, 2015; Wang et al., 2019). To gauge the extent of ILS in our dataset, we performed concordance analyses using two different approaches. First, we used ASTRAL (Zhang, Rabiee, Sayyari, & Mirarab, 2018) to assess quartet support for the branch (i.e. the percentage of quartets in single‐locus trees which agree with the inferred concatenated tree). As an alternative, we used PhyParts (Smith, Moore, Brown, & Yang, 2015) to gauge the agreement in terms of internal branches between the individual locus trees and the concatenated tree. With both measures, we observed low concordance for all internal branches within the bird clade, indicating that individual trees are generally very different from the average (concatenated) tree (Figure S6).

Collectively, these analyses indicate that placement of owls in the Avian tree of life remains uncertain. This is due to the very high level of discordance among individual loci—likely due to a combination of hard‐to‐resolve, short internal branches, and Incomplete Lineage Sorting. Furthermore, because of the uncertainty in the true placement of owls, we are unable to conclusively assess the impact of genome quality on the inferred owl phylogeny.

## 3 DISCUSSION

The assembled genome of the European barn owl integrating Illumina and PacBio technologies shows improved properties compared with the Illumina‐only‐based American barn owl assembly. Out of the 1.59 Gbp (C‐value‐derived genome size), 1.22 Gbp could be assembled. Similar results were found for other raptors, a Strigiformes, the Northern spotted owl (*Strix occidentalis caurina*), the peregrine falcon (*Falco peregrinus*), and the cinereous vulture (*Aegypius monachus*) for which the assembled genome size was estimated to be 1.26, 1.22, and 1.13 Gbp for C‐value genome size of 1.5, 1.42, and 1.56 Gbp, respectively (Chung et al., 2015; Hanna et al., 2017; Zhan et al., 2013). Thus, while still incomplete, the European barn owl has an extra 7% assembled compared with the American barn owl. Some of the assembly metrics are as good as those of zebra finch and collared flycatcher. The genome completeness analyses are close to the well‐assembled chicken genome (Table 2). In addition, the identification of the scaffolds belonging to the Z chromosome and their mapping to the zebra finch indicates that the assembled genome has few miss‐assemblies and few rearrangements (Figure 4b). The cumulative size of the identified Z chromosome scaffolds of 70 Mb is comparable to that of other bird genomes, the Z chromosome being 82.3 Mb in the chicken (Hirst, Major, & Smith, 2018), 73 Mb in the zebra finch (Rutkowska, Lagisz, & Nakagawa, 2012), and 59.7 Mb in the collared flycatcher (Kawakami et al., 2014). In addition, the barn owl genome appears to have few rearrangements or miss‐assemblies when compared to the zebra finch genome. High chromosome synteny is observed in most birds, except in the Falconiformes and the Psittaciformes containing high levels of interchromosomal rearrangements (O'Connor et al., 2018).

The GC content impacts the short‐read sequencing, as has been shown previously (see for example Botero‐Castro et al., Figure 5b, (Botero‐Castro et al., 2017). Fifteen percent of genes present in most vertebrate lineages and thought to be missing in the avian genomes (Lovell et al., 2014) are located in very GC‐rich mini‐chromosomes (Bornelov et al., 2017; Botero‐Castro et al., 2017). For instance, the recently sequenced *Leptin* gene in chicken and duck has an overall GC content of 67 and 74%, respectively (Seroussi et al., 2016), in the range of the various avian leptin genes (GC: range 66% to 82%) and higher than in mammals (56 and 58% in human and rat, respectively. The high GC content in exons of the European barn owl is also evident in the set of the 57 Sanger sequenced genes analyzed. Why some regions are so highly GC‐rich in birds is an open question. One reason may be for regulatory functions since the 5' ends of the genes are GC‐richer and contain CpG islands in their transcription start site and promoter that could be regulated by methylation. Another specificity of these new sequenced GC‐rich avian genes is that they appear to be associated in clusters in mini‐chromosomes (Bornelov et al., 2017). Thus, due to technical difficulty many genes remain non‐ or partially sequenced in birds (Botero‐Castro et al., 2017). A recent study estimated the proportion of missing genome in typical bird assemblies at ~20% (Peona et al., 2018). However, in the European barn owl genome we retrieved genes missing in chicken or other birds, indicating that the genome is of relative high quality and completeness.

We identified 38,000 protein‐coding genes in the genome of the European barn owl. In the chicken genome version 5, Warren identified 19,119 protein‐coding genes and 6,839 noncoding genes (Warren et al., 2017) and Kuo 60,000 different transcripts using long‐read RNA sequencing (Kuo et al., 2017) suggesting that the range of expected genes and transcripts in birds is as high as in mammals. Concerning the comparison of the predicted proteins with BUSCO orthologs, few papers have reported it. It was the case for *Coturnix japonica, Colinus virginianus, Bambusicola thoracicus, Meleagris gallopavo,* and the chicken annotations that recovered 46%, 54%, and 45%, 80% and 90%, respectively, of complete BUSCO vertebrate orthologs (Tiley, Kimball, Braun, & Burleigh, 2018). This places the European barn owl and its annotation (with up to 74% of protein retrieved), in the upper hand of the annotated avian genomes.

Using the European barn owl genome and annotation, phylogenetic analyses position the barn owl as an outgroup to the Coraciimorphae and separated from the Accipitrimorphae. This contrasts with the position of the same species obtained with the American barn owl annotation, which places it as a sister group to the Accipitrimorphae (Figure 6) as proposed by Jarvis (Jarvis et al., 2014; Suh et al., 2015) and the proposed position by Prum et al. (2015).

Although genome quality measures (e.g., NG50, BUSCO measure) are indicative of a more complete and accurate European barn owl genome, the benefit of this for phylogenetic inference remains largely inconclusive. Resolving the difficult placement of owls on the Avian tree of life will likely require denser taxon sampling of the Strigiformes.

## 4 CONCLUSIONS

Although there is room for improvement when compared to the genomes of model species, the current assembly represents a significant advance from previous genomic data in barn owls. Next step would be to complete our genome to get continuous chromosomes and to characterize the missing parts. As it stands, the European barn owl genome will be an extremely valuable resource for carrying further analyses, both at the structural, functional, and evolutionary level.

## 5 MATERIALS AND METHODS

### 5.1 Extraction of genomic DNA and libraries preparation

Genomic DNA was extracted from ‐ 80°C frozen blood sample of a young male barn owl (M026801) using MagAttract HMW DNA Kit (Qiagen). In total five extractions quantified on a Qubit Fluorometer (ThermoFisher Scientific) yielded each between 1 and 6 µg of genomic DNA of a length between 35 and 50 kb (Fragment analyzer, Advanced Analytical, Labgene). In order to obtain a high‐quality de novo assembly, we combined libraries generated from standard short insert paired‐end libraries with that from mate‐pair libraries and PacBio long sequencing. Two mate‐pair libraries of 2 and 5 kb were prepared and sequenced by Fasteris (Fasteris), three TruSeq paired‐end libraries of 180 and twice 500 bp were prepared and sequenced at the Genomic Technologies Facility (GTF, University of Lausanne). The same high molecular weight DNA was used for PacBio libraries at the GTF. For PacBio, the DNA was sheared in a Covaris g‐TUBE (Covaris) to obtain 20 kb fragments. Then, the DNA size distribution was checked on the Fragment Analyzer. 5 µg of the sheared DNA was used to prepare a SMRTbell library with the PacBio SMRTbell Template Prep Kit 1 (Pacific Biosciences) according to the manufacturer's recommendations. The library was sequenced on 37 SMRT cells with P4/C2 chemistry and MagBeads on a PacBio RSII system (Pacific Biosciences) at 240 min movie length.

### 5.2 RNA extraction and transcriptome

Six tissues (liver, heart, kidney, testis, growing back feathers, and thalamus) were sampled from a 57‐day‐old nestling barn owl (M026830) from our wild population located in Switzerland and that had died for unknown reasons in the nest in our presence. Dissected tissues were immediately transferred to dry ice and kept at −80°C for long‐term storage. Between 20 and 120 mg of each tissue sample were bead‐homogenized at 4°C with Trizol (Life Technologies) in a MagNA Lyser (Hoffmann‐La Roche Ltd., Basel, Switzerland) at 6,500 rpm for 3 × 30 s. Total RNA was extracted from tissue homogenates with RNeasy kit (Qiagen) and eluted in 50 μl water. Five μl aliquots of total RNA samples was diverted to assess quantity and quality with a Qubit fluorometer (Life technologies) and Fragment analyzer (Advanced analytical, Labgene), respectively. Only total RNAs with a RQN >8.0 were used to prepare 6 KAPA stranded mRNAseq Libraries (KapaBiosystems, Roche) at the GTF (GTF, University of Lausanne).

### 5.3 Genome assembly

The five different short‐read libraries sequenced on an Illumina HiSeq platform resulted in 605 million paired‐end reads and in 106 million mate‐pair reads of size 2 × 100 bp. In total, 143 Gbp were sequenced (Table 1). Multiple genome characteristics (genome size, heterozygosity) were estimated using a kmer counting approach (Jellyfish (http://www.cbcb.umd.edu/software/jellyfish/) combined with GenomeScope (http://qb.cshl.edu/genomescope/) (Pearson & Lipman, 1988; Vurture et al., 2017) with a kmer size of 21 and run on the three paired‐end libraries later used for contig assembly. The heterozygosity value is derived from the smaller peak at half of the expected coverage (75% for the Illumina reads) (Figure S3).

After read quality control (Andrews, 2010), reads were assembled using SOAPdenovo (Luo et al., 2012) (version 2.04.240). While paired‐end reads were used for assembly and scaffolding, mate‐pair reads were used solely for scaffolding. We tried various kmer sizes. The assembly based on kmer 47 outperformed the other assemblies in the number of scaffolds and N50 metric and was retained. Gaps within this assembly were closed using GapCloser [8] reducing the proportion of Ns from 8.8% to 1.4%. The short‐read base assembly was further scaffolded using 37 P4C2‐chemistry PacBio smrt cells and PbJelly (English et al., 2012) (version 14.4) with default options. A second round of gap closing was run reducing the proportion of Ns to 0.79%.

### 5.4 Quality assessment

Quality and completeness of the assembly was assessed by computing assembly metrics, such as N50, NG50, and longest contig, by mapping back the raw paired‐end Illumina reads with bowtie2 (Langmead & Salzberg, 2012) (version 2.2.4) and by searching for universal single‐copy orthologs using BUSCO (Simao et al., 2015) (version 2) with the avian BUSCO set and default parameters. The assembly metrics and BUSCO results were compared with the assemblies of the American barn owl, the zebra finch, the flycatcher, and the chicken.

### 5.5 Transcriptome assembly

The transcriptome was assembled using the six libraries prepared with the Kapa stranded mRNAseq Library Preparation kit (Roche) to minimize the sequencing bias for high GC content regions. Libraries were sequenced using the Illumina HiSeq platform at the GTF resulting in total 192 million reads of size 2 × 100 bp summing up to 34 Gbp per tissue (Table S4). The reads were assembled using Trinity (Haas et al., 2013) (version 2014.07.17) with default parameters and the option trimmomatic for quality trimming reads before assembling them. The resulting transcriptome consisted of 421,658 contigs ranging from 201 to 21,648 bp with a mean of 808 bp and a median of 382 bp. The transcriptome (Tyto_Alba_DEE_transcriptome.fasta) was filtered for transcripts with homologies to known proteins, by first extracting long open reading frames (ORFs) followed by a blast and a pfam search to known proteins (Haas et al., 2013).

### 5.6 Repeat annotation

The evaluation of repeats and low complexity regions were assessed using RepeatModeler version 1.11.0 (Smit & Hubley, 2008–2015) and RepeatMasker version 4.0.7 (Smit et al. 2013–2015) with default parameters. RepeatMasker was run with RMBlastn version 2.6.0+ for the genome of the American and European barn owl, the zebra finch, the flycatcher, and the chicken individually.

### 5.7 Gene prediction and annotation

Genes were predicted using Augustus 3.0.1 (Stanke, Schoffmann, Morgenstern, & Waack, 2006) with a custom trained model. We trained the gene prediction models for Augustus using the transcriptome data and evaluated the predictions on a set of 57 Sanger sequenced genes. However, because the transcription start sites (and first exon) were often missing, the best model was obtained by mapping the Uniprot reference proteome of the chicken onto the European barn owl assembly with BLAT (Kent, 2002) (version 3.4), then the resulted matches were translated in Augustus‐compatible training sets using Scipio (Keller, Odronitz, Stanke, Kollmar, & Waack, 2008) (version 1.4.1) and Augustus provided scripts. Genes were predicted for our assembly on scaffolds larger than 500 bps (options: alternatives‐from‐evidence = false, alternatives‐from‐sampling = false, noInFrameStop = true, UTR = off).

### 5.8 Proteome comparison

The predicted proteins of the chicken Augustus assembly and the NCBI predicted proteins of the American barn owl genome were compared in a global‐global fashion against the chicken reference proteome from Ensembl (ensembl release 88, 30,252 supported protein sequences) using ggsearch (Pearson & Lipman, 1988) (version 36.3.5e, options: ‐b 1 ‐d 0 ‐E 1e‐5 ‐k 1 ‐m 8).

We also assessed the completeness of the proteome and compared it to the other proteomes by searching for universal single‐copy orthologs using BUSCO (Simao et al., 2015) (version 2) with the metazoa BUSCO set and default parameters.

### 5.9 Comparison of the European and the American barn owl genome

To detect genome structural variants between American and European barn owls, we did not compare the two assemblies directly to each other because their quality differs, and consequently it would be difficult to distinguish biologically relevant differences from technical artifacts. Instead, we mapped the raw Illumina reads used for both assemblies to the European barn owl assembly using bowtie 2 (Newman et al., 2005). A total of 102,000,000 reads (96.2%) of the American barn owl were mapped on the European barn owl assembly and 206,000,000 reads (95.9%) of the European barn owl were mapped on the European barn owl assembly. Since the coverage was highly variable, we computed the ratio for bins of 1kb and potential interesting sites were searched visually. By comparing the two coverages to each other, it was possible to find real biological differences between the two sequenced barn owls. This was true if the Illumina libraries were prepared and sequenced in a similar way. We expected that the read coverage ratio of European barn owl reads over the sum of American and European raw reads would be equal to 0.5 when the coverages are corrected for library size. Deviating ratios may be indicators of duplications and deletions.

### 5.10 Comparison of the European barn owl and the zebra finch

To assess the chromosomal structure of the European barn owl assembly we aligned the scaffolds to the zebra finch genome using blast, option MegaBLAST and word size 48. Hits were subsequently filtered for a minimal size of 1,000 bp. The zebra finch genome was one of the closest related species for which the genome was assembled at the chromosomal level. Although this species counts 40 pairs of chromosomes and one germline restricted chromosome (Biederman et al., 2018; Torgasheva et al., 2019), only 37 chromosomes and scaffolds were assembled in Ensembl zebra finch karyotype (Figure S1).

### 5.11 Copy number variation quantification by qPCR

Genomic DNA of 4 female and 4 male European Barn owls of Switzerland and 4 female and 3 male American Barn owls of the Museum of Vertebrate Zoology, Berkeley, California, USA were extracted as described (Uva et al., 2018). The DNA quality was assessed on 0.8% agarose gels and quantified with the Qubit (ThermoFisher Scientific). All DNA samples that appeared not fragmented on gel were diluted to 10 ng μl^−1^.

qPCR primers pairs and probes were designed so that one primer and probe pairs amplified DNA of the predicted duplicated region and one of the upstream region with the following sequences: for the contig 97, the RAB32 gene was used: the control region in the exon 1: RAB32_ex1_111F: GTACGTGCACCAGCTCTTCTC, RAB32_ex1_191R: CTGTCCCAGTTGATGACTTTGA and the probe fluorescein‐labeled probe RAB32_ex1_148Fam‐Q1: ACCATCGGGGTGGATTTCGCTC, for the expected duplicated region: RAB32_ex2_46F: AGGCAGTTGGTGCTTTTGTGGT, RAB32_ex2_179R: TGCAAGAAGAACAGCAGGGATG and the probe RAB32_ex2_74Fam‐Q1: TGTCACAAGAGGCTCCACTTTTGAGGCTG.

Each qPCR was set up with different probe and primers concentration and various DNA concentrations to get similar qPCR efficiency. Two μl of Swiss and American genomic DNA was tested in duplicates on a ABI7500 with 1× TaqMan GeneExpression Mix (Life Technologies, Thermo Fischer Scientific, Switzerland), in 20 µl with 0.15 μM of each primer pairs and probes. When Ct values for duplicates differed in more than 0.3 Ct the result was not considered. The standard curves were used to calculate the number of copies relative from the Ct values and the ratios of the expected duplicated to the control region was calculated.

### 5.12 Avian lost genes

The same mammalian and lizard lost proteins described by Warren et al. (Tables S3 and S4 in Warren) (Warren et al., 2017) namely the 129 lost proteins in chicken but found in other birds (Table S3a) and the 232 proteins found in no avian genome (Table S3b) were searched in Ensembl BioMart for their human Uniprot Swiss‐Prot entries (one of the 129 lost proteins set was not retrieved with BioMArt) and aligned them (tblasn 2.7.1+, evalue < 1e‐10) against the barn owl transcriptome (Tyto_Alba_DEE_transcriptome.fasta). Then, the recovered transcripts were reciprocally aligned (blastx 2.7.1+, with E‐value <10^–9^, only the entry with the best alignment score was selected) against Swiss‐Prot to ensure that the right protein was found and controlled manually for Swiss‐Prot entries with the correct name or correct gene description, if the first hit was of another species.

### 5.13 Phylogenetic tree inference

We compiled five datasets to assess the impact of a new annotation on the positioning of *Tyto alba* in the bird tree of life.

As a starting point for all datasets, we selected 9 species from the May 2016 OMA database (Altenhoff et al., 2018) and further 7 species including the American NCBI annotation of the American barn owl were retrieved from http://avian.genomics.cn/en/jsp/database.shtml (protein sequences from the GigaDB annotations, Table S5) and our annotations of the European barn owl (*Tyto alba alba*) set of predicted protein sequences. These included all available saurian species at that time available in the OMA database. Moreover, this included 2 mammals and *Xenopus tropicalis* as outgroup. In total this dataset included 17 species. We inferred the orthologous relationships between the species using OMA standalone (Altenhoff et al., 2019). Phylogenetic marker genes were then selected using a threshold of minimum 16 species included in an orthologous group (OG). With this threshold, we obtained 2,578 OGs.

From this, we constructed three datasets. The first two datasets (i, ii) contained either American or European barn owl proteomes. The third dataset (iii) contained both proteomes.

As for datasets (iv) and (v), we added three more species of owls to dataset (i): *Strix occidentalis caurina, Bubo blakistoni*, and *Athene cuniculata*. Details are available in Table S5. OMA standalone was run again to predict the orthology. Phylogenetic marker genes were then selected using a threshold of minimum of 19 species, resulting in 1,053 orthologous groups (OGs).

For all datasets, OGs were then aligned using Mafft v7.310 (Katoh & Standley, 2013) (par: ‐maxiterate 1,000 ‐‐localpair), concatenated and trimmed using trimAI v1.4.1 (Capella‐Gutierrez, Silla‐Martinez, & Gabaldon, 2009) with the gappyout parameter. Phylogenetic trees were inferred using RaXML‐NG v0.9.0 (Kozlov, Darriba, Flouri, Morel, & Stamatakis, 2019) (‐‐model LG + G8 + F ‐‐seed 15,826 ‐‐all ‐‐bs‐trees 100). The differences in topology were visualized using Phylo.io (Robinson, Dylus, & Dessimoz, 2016).

Assessment of incomplete lineage sorting as source for topological variation was performed with ASTRAL v5.6.3 (Zhang et al., 2018) and PhyParts v0.0.1 (Smith et al., 2015) on the small dataset. As input data we used the 2,578 OGs that were aligned with Mafft v7.310 (par: ‐maxiterate 1,000 ‐‐localpair), and gene trees were computed with IQTREE v 1.6.11 (Nguyen, Schmidt, Haeseler, & Minh, 2015) (par: ‐nt 1 ‐mem 2G ‐seed 12,345 ‐m LG) and the two reference trees (Figure S6).

## AUTHOR CONTRIBUTION

Jérôme Goudet, Alexandre Roulin, and Ioannis Xenarios designed the research. Anne‐Lyse Ducrest extracted genomic DNA and total RNA. Mélanie Dupasquier prepared the libraries. Samuel Neuenschwander, Emanuel Schmid‐Siegert, and Marco Pagni assembled the genome, compared it to other genomes and participated in the genome annotation. Clément Train, David Dylus, Alex Warwick Vesztrocy, Yannis Nevers, and Christophe Dessimoz performed the phylogenic analysis. Anne‐Lyse Ducrest searched for lost genes. Anne‐Lyse Ducrest and Luis M. San‐Jose studied GC composition. Anne‐Lyse Ducrest, Alexandre Roulin, and Jérôme Goudet wrote the manuscript and all authors contributed to the editing and approved the final manuscript.

## Data Availability

The raw Illumina (genomic and RNAseq) and PacBio (genomic) reads and the genome and transcriptome assemblies have been deposited in European Bioinformatics Institute European Nucleotide Archive database under accession number PRJEB32835 (secondary accession number: ERP115928).
