# Supplementary material for: New genome assembly of the barn owl (Tyto alba alba)
Source: Ecol Evol. 2020 Feb 19;10(5):2284–98. doi: 10.1002/ece3.5991 (PMC7069322; doi:10.1002/ece3.5991)
Supplement: Supplementary file 1 [file ECE3-10-2284-s001.docx]

**Appendix Figures**


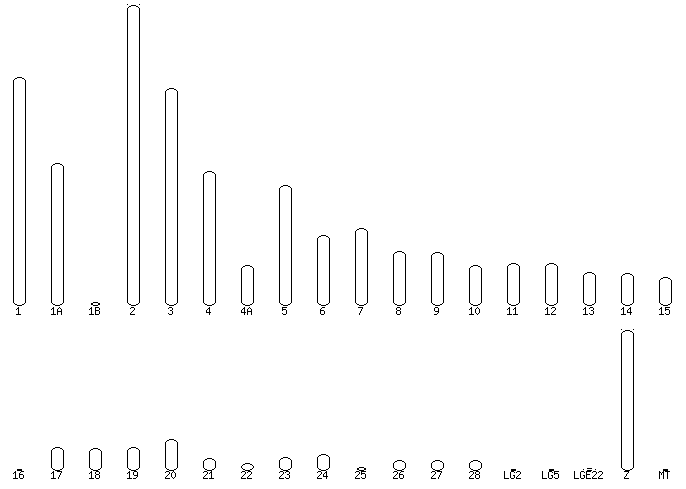


**Figure S1**: Karyotype of the zebra finch

**Figure S2**: Circos plot that compares the different zebra finch chromosomes (black) to the European barn owl scaffolds (green). The coverage of American and European barn owl raw reads is shown in blue and red, respectively.


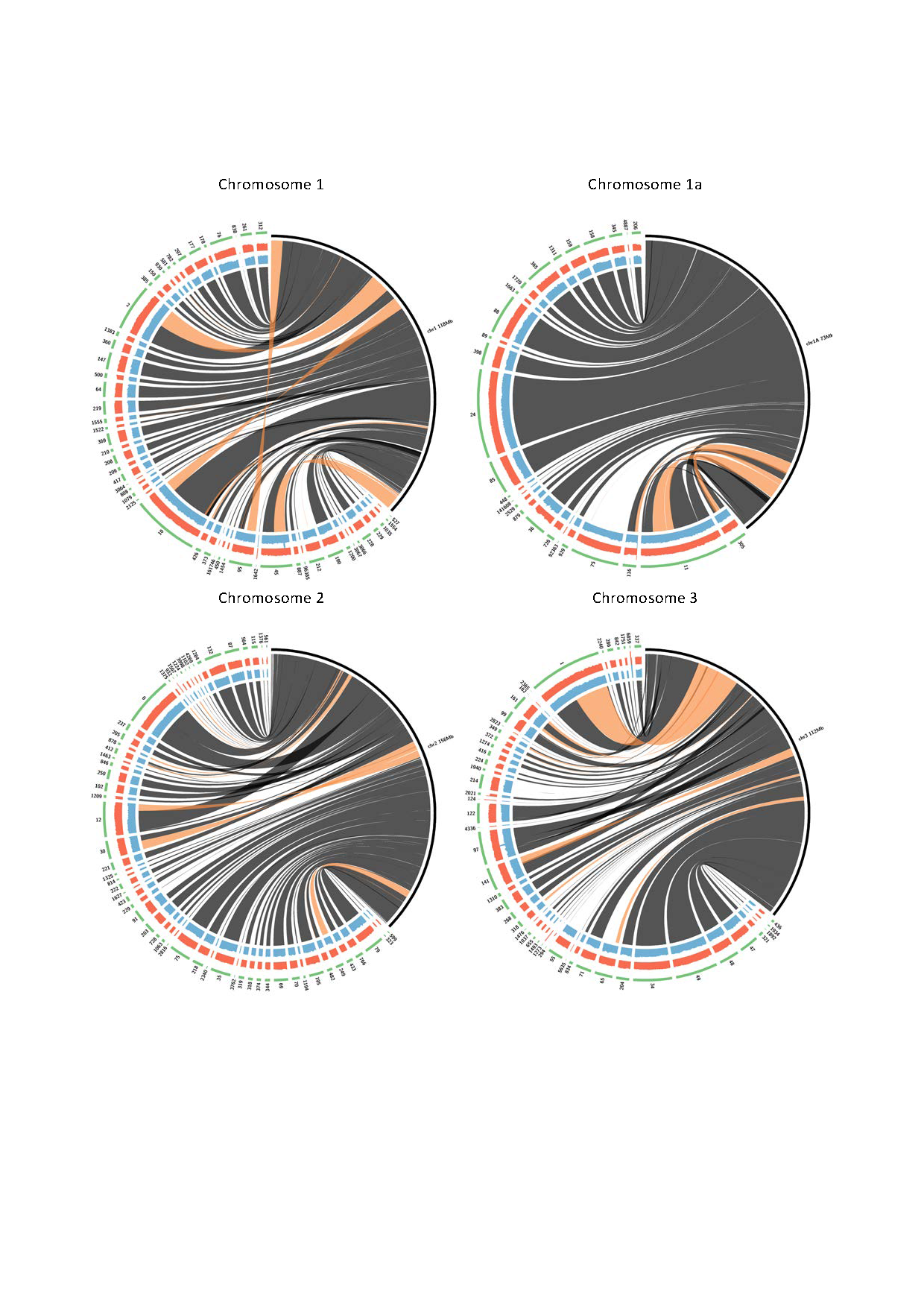

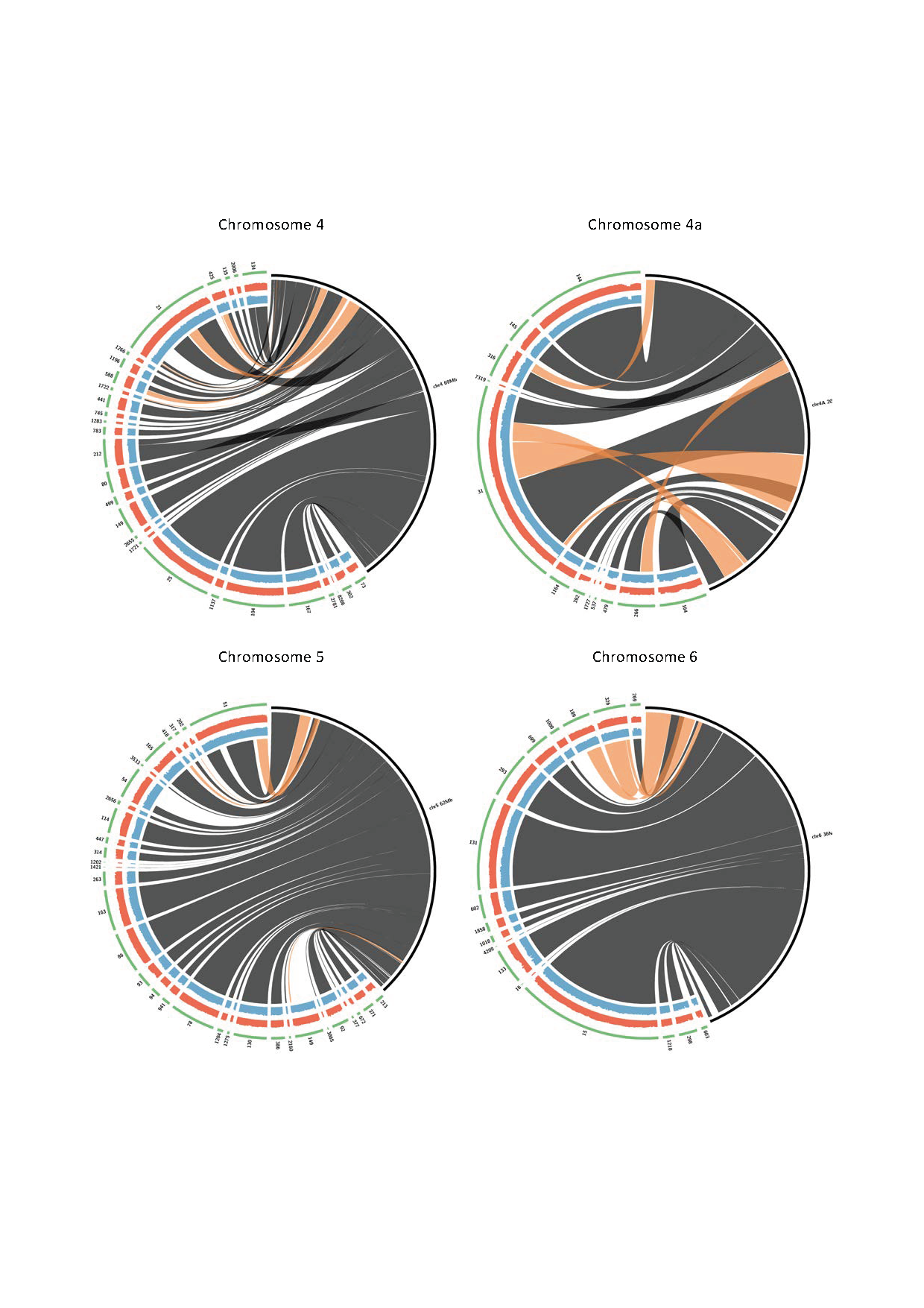

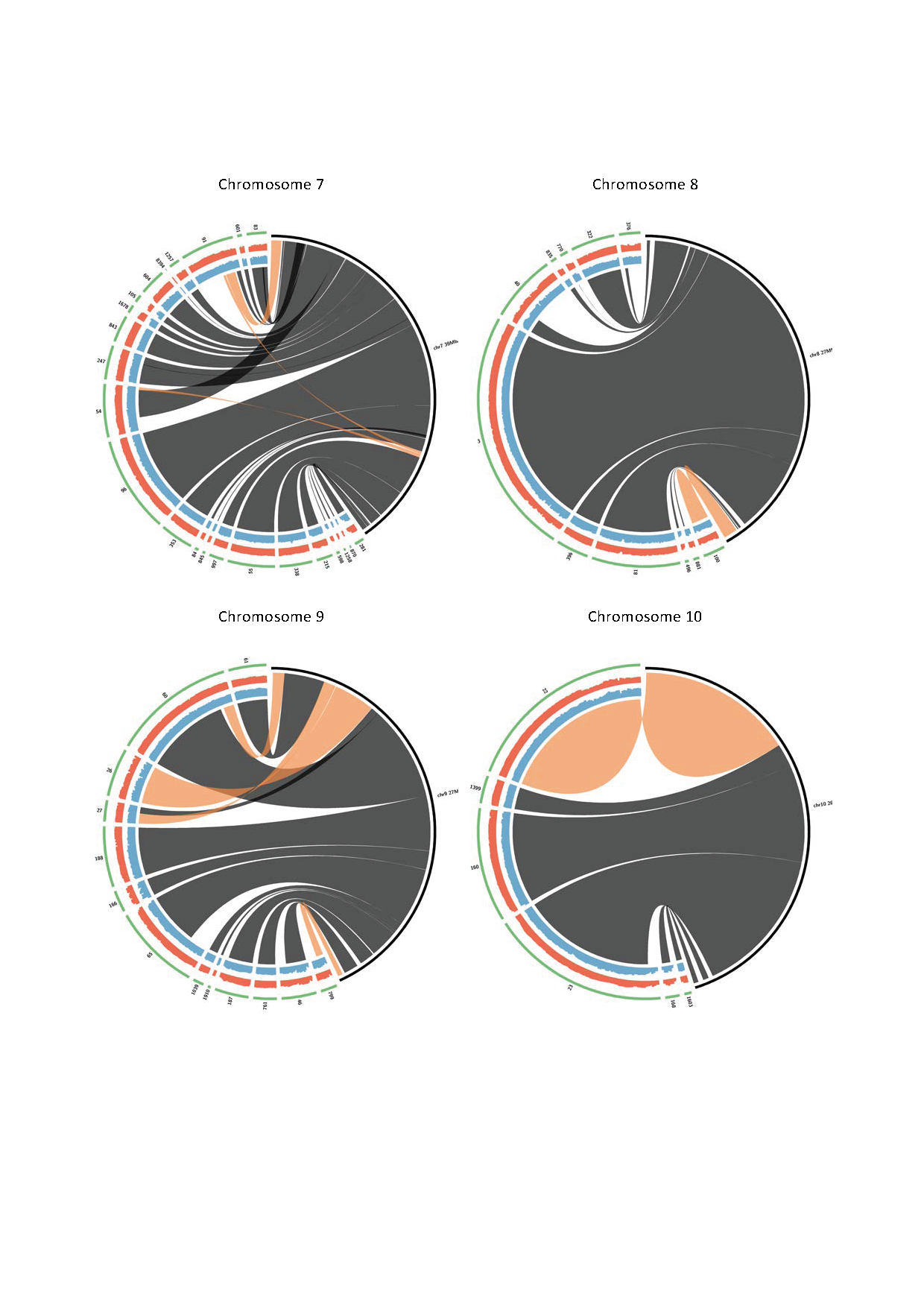

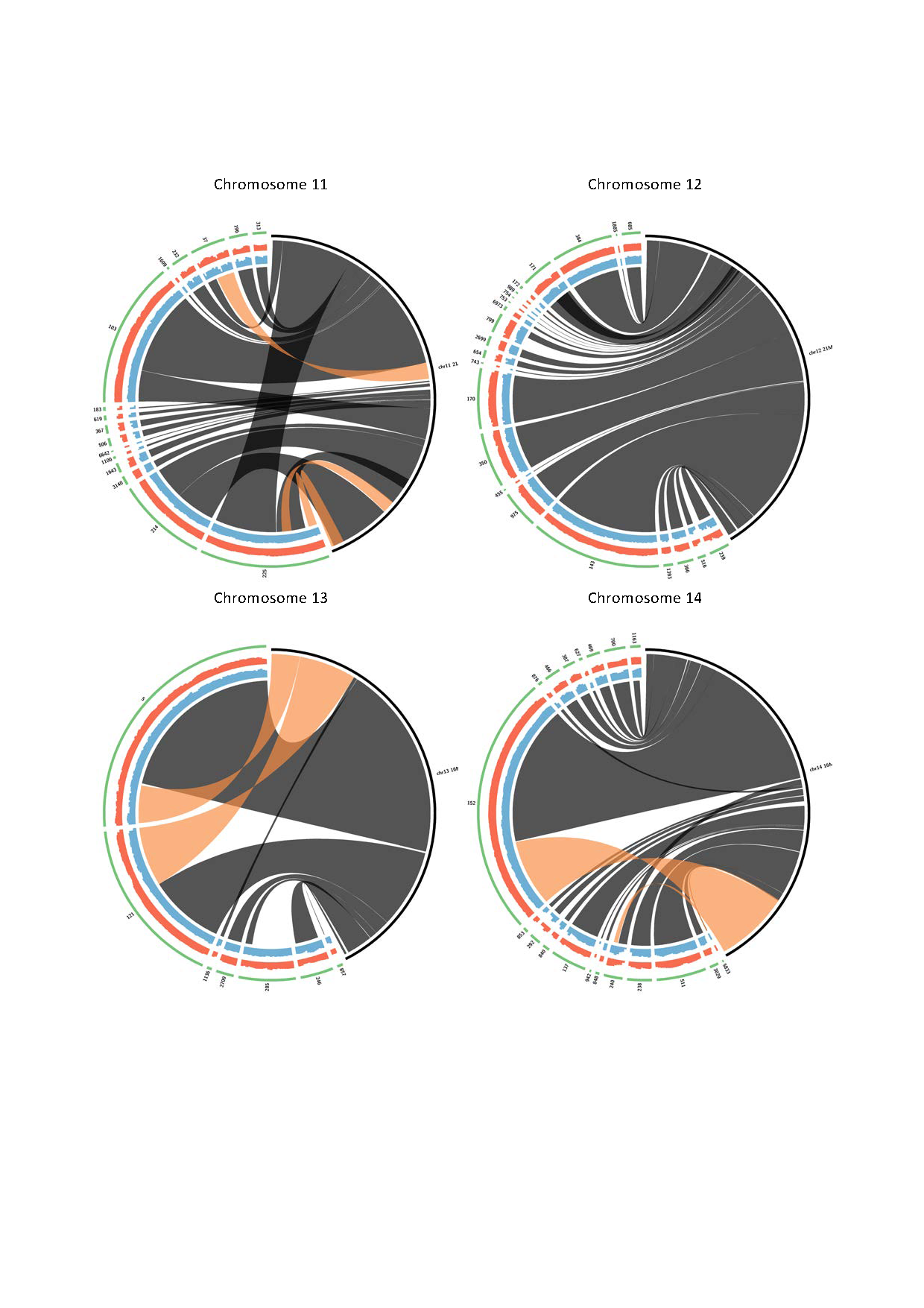

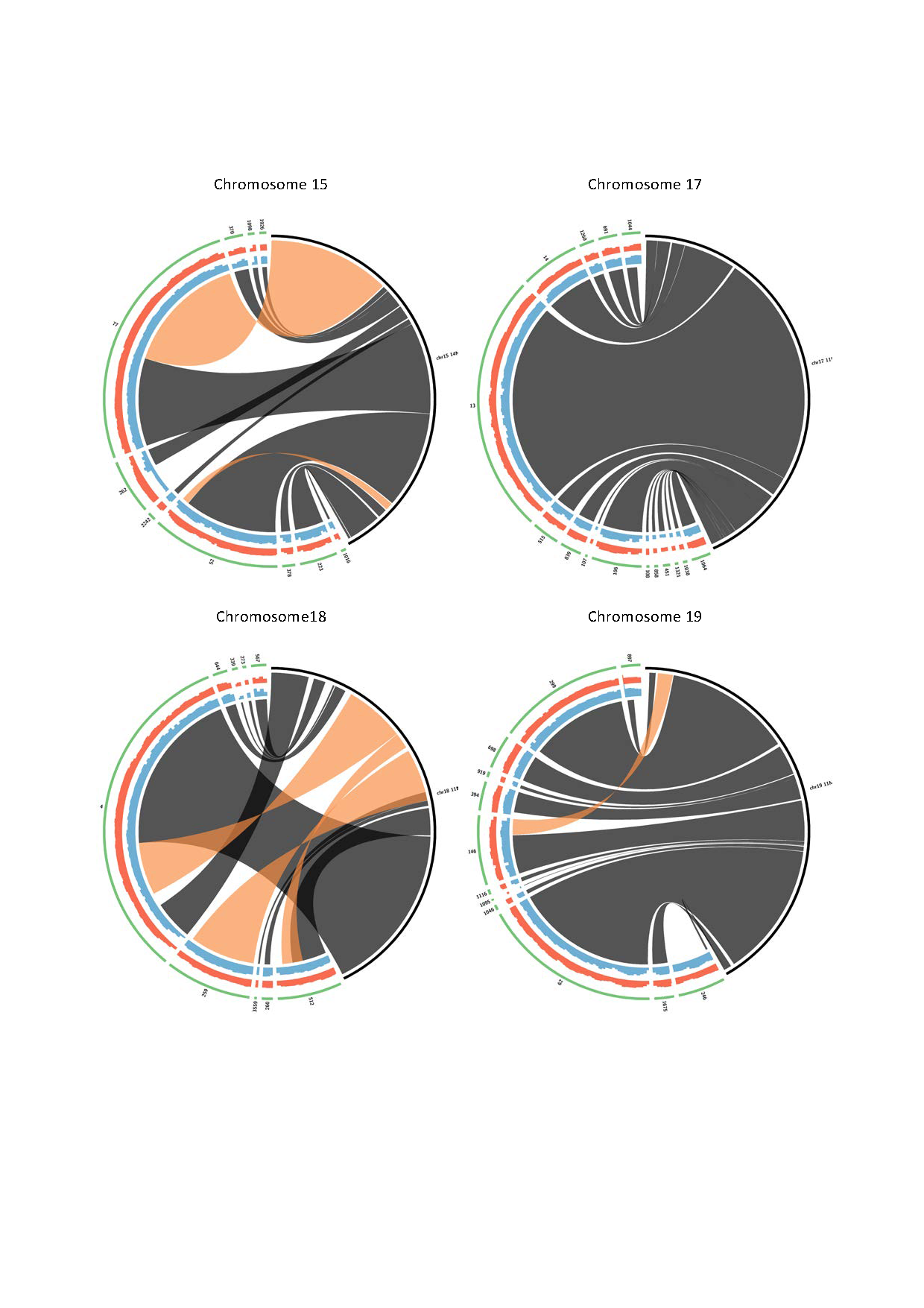

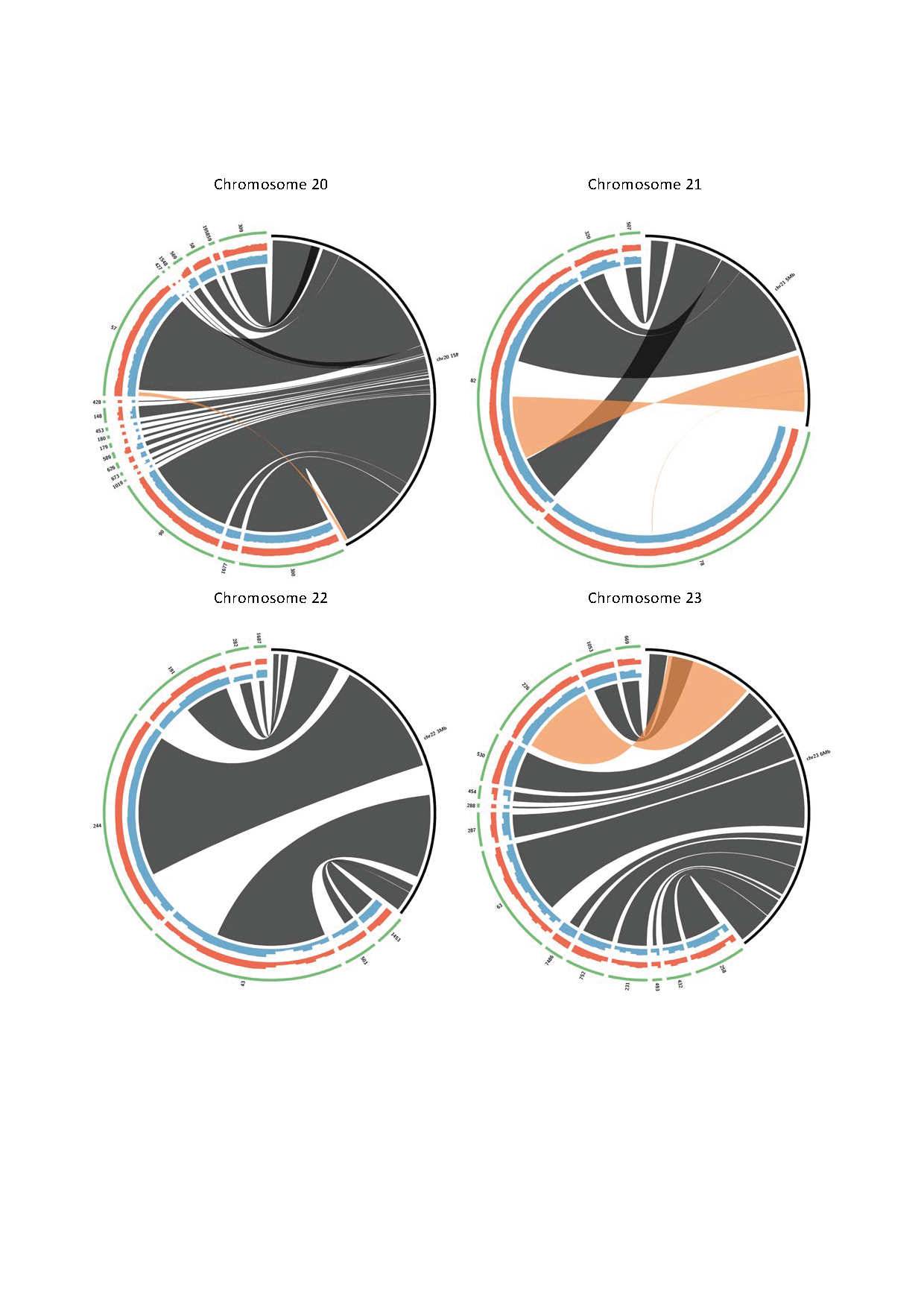

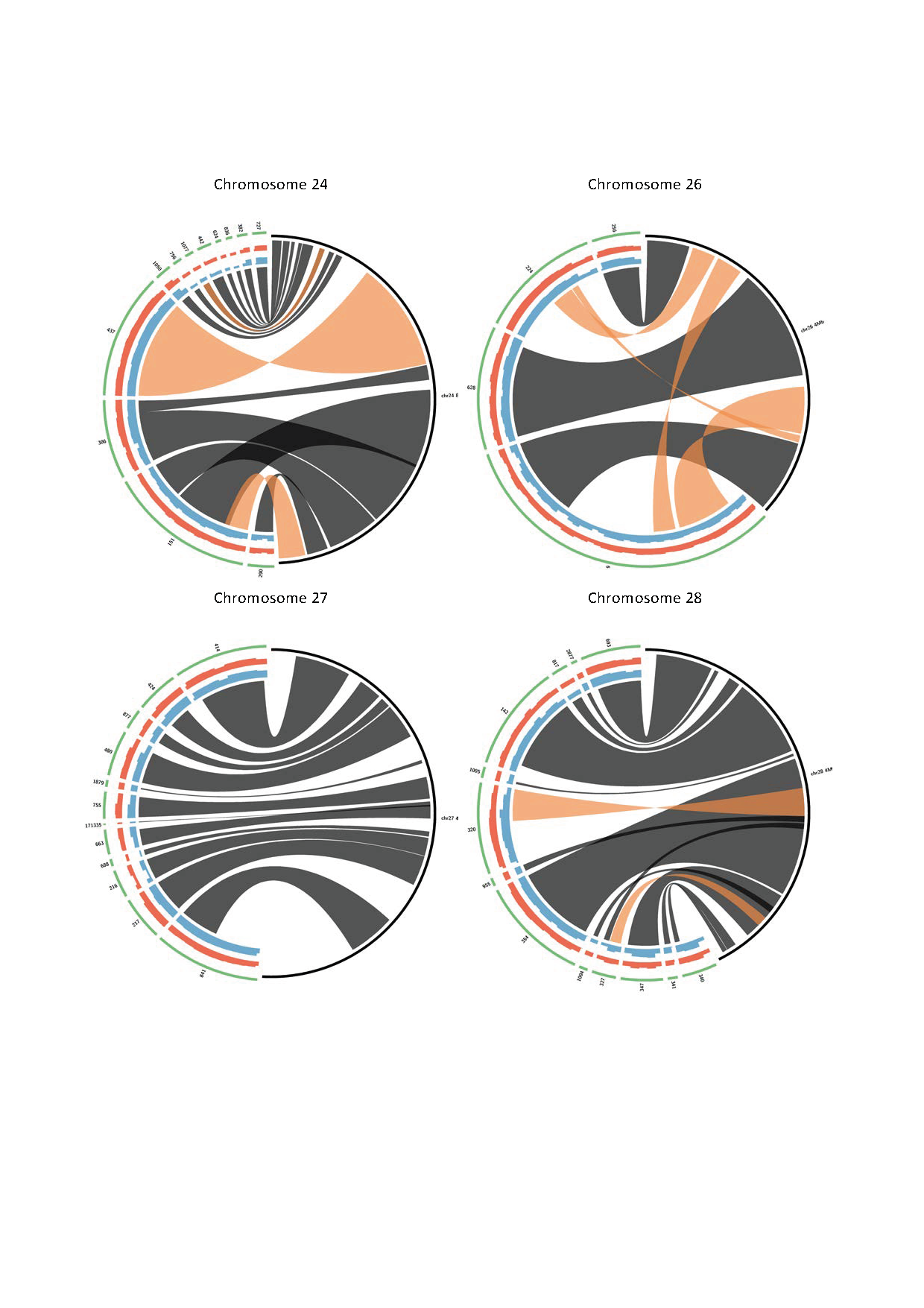

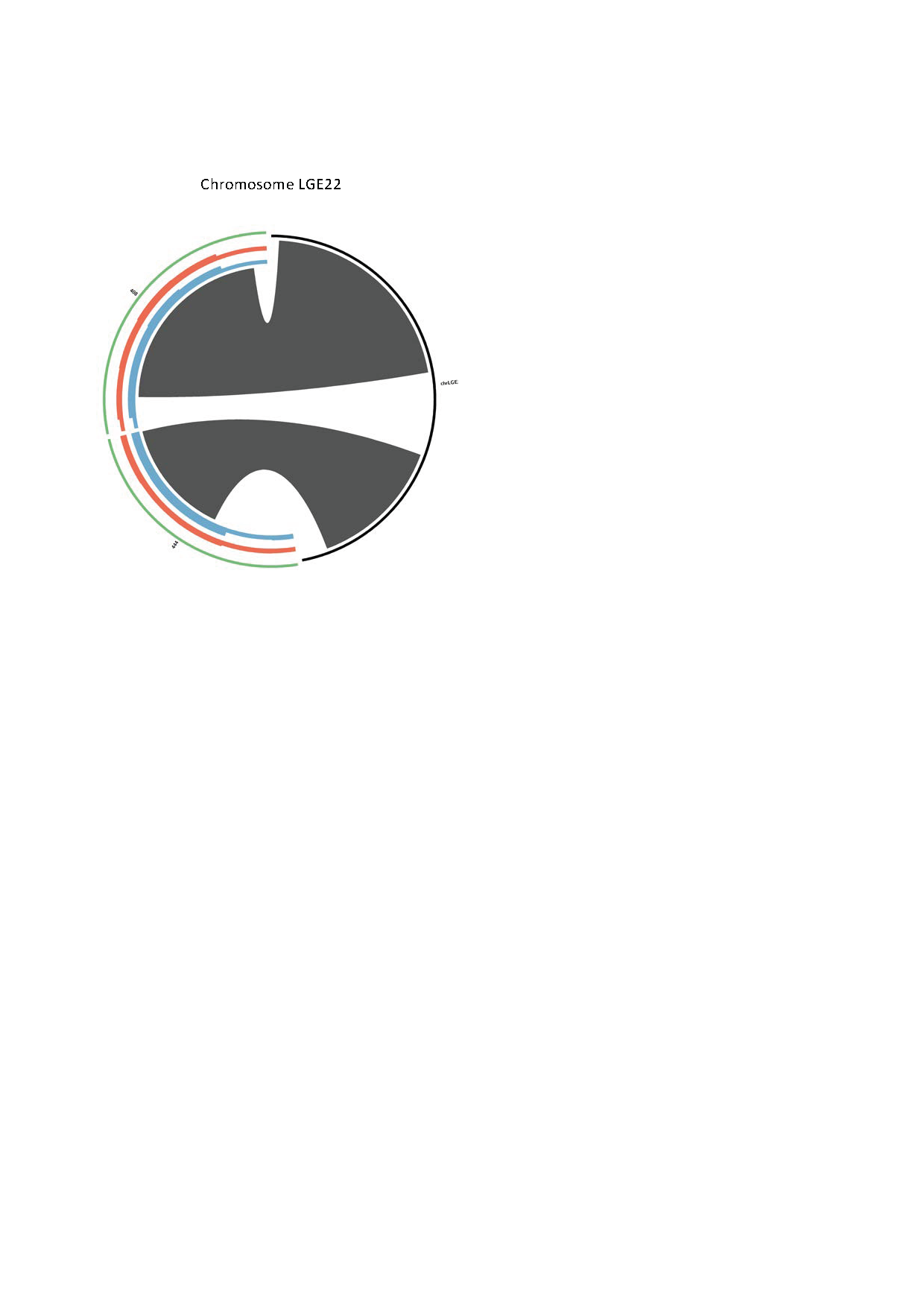


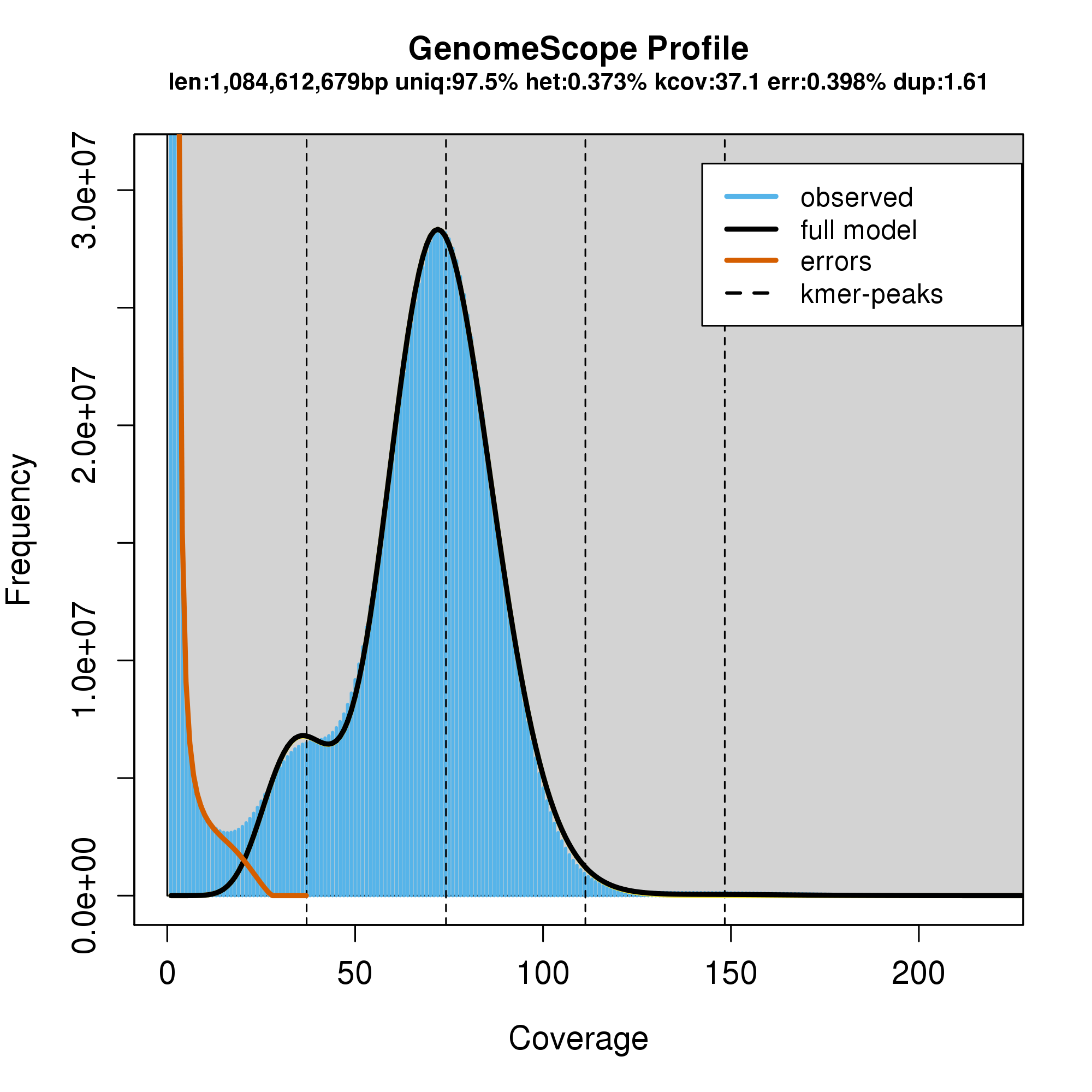


**Figure S3**: Genome profile with a 21-kmer count distribution of the Illumina paired-end libraries of the European barn owl. The height of the small blue peak is proportional to the heterozygosity rate. The orange line shows the sequencing errors present with low coverage. and the yellow line show the unique sequences of the Illumina paired-reads.


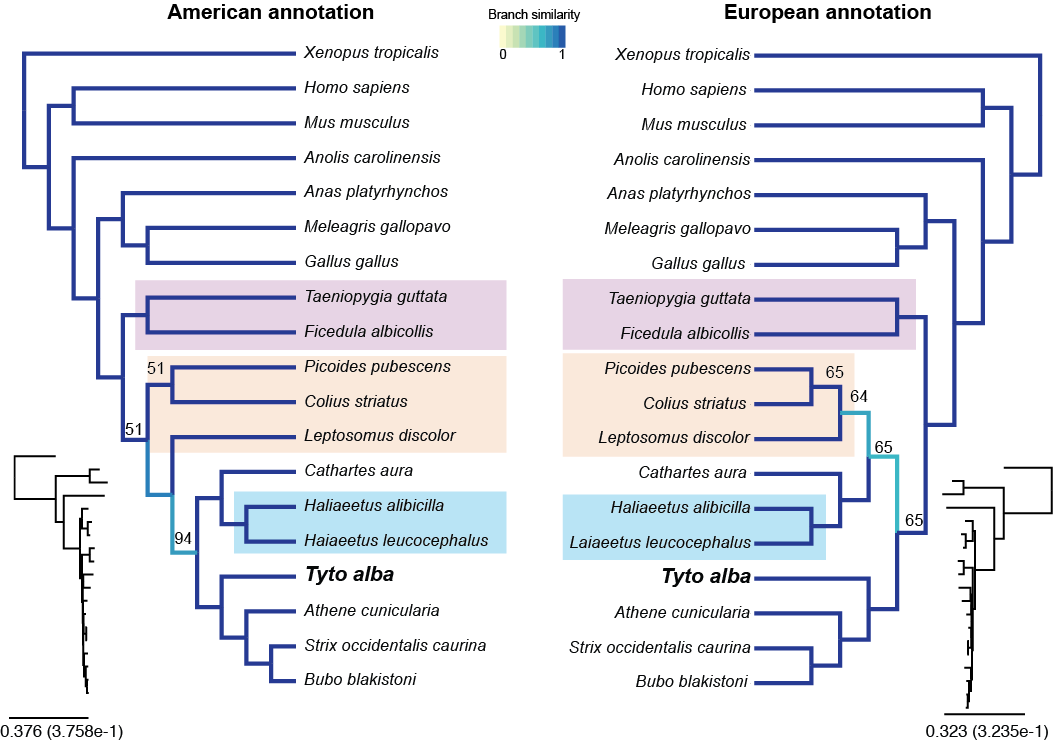


**Figure S4**. Avian phylogenetic trees based on the American and European barn owl proteins predicted with the American and European barn owl annotations and 3 other owl species. Depending on the dataset, the position of owls varies on the tree. Left tree used the protein predictions produced by the American annotation, right tree uses the protein predictions produced in this work. Shades of blue show positions in which differences in topology are detected. Nodes without number have a bootstrap support of 100 %; Small trees on the sides show the real branch lengths. The purple background represented the group of Passerimorphae, the orange the Coraciimorphae and the light blue the Accipitrimorphae.


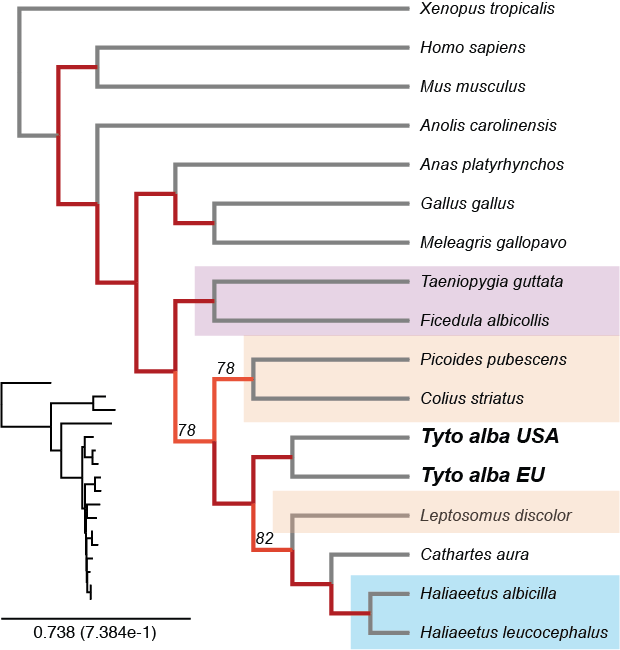


**Figure S5**. Avian phylogenetic trees based on the American and European barn owl proteins predicted with the American and European barn owl annotations. Shades of red show bootstrap support with dark red being 100. Nodes without number have a bootstrap support of 100; Small trees on the sides show the real branch lengths. The purple background represented the group of Passerimorphae, the orange the Coraciimorphae and the light blue the Accipitrimorphae.


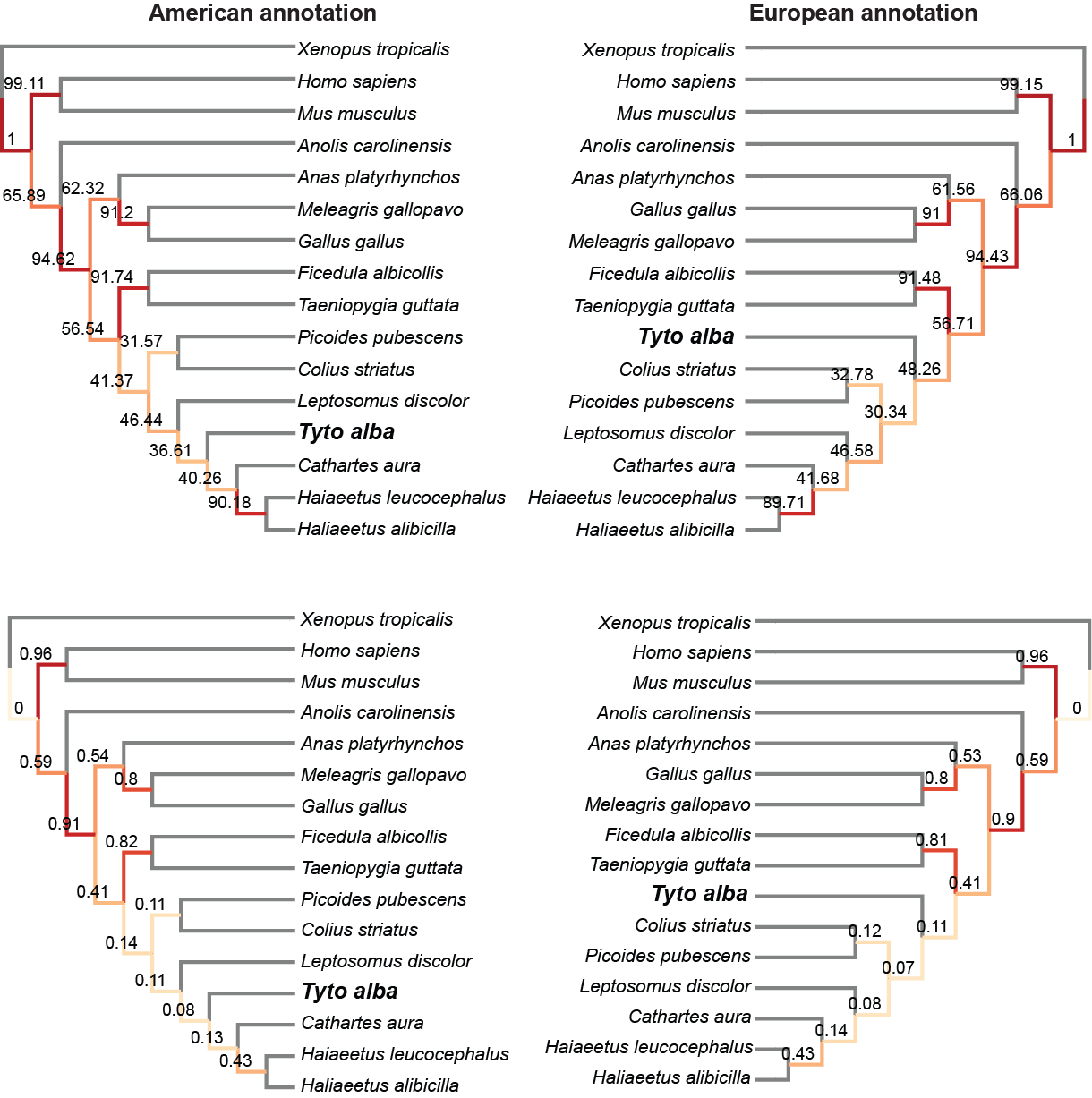


**Figure S6**. Low concordance of internal branches. Top row shows ASTRAL analysis with quartet support on branches with dark red having support of 100. Bottom row shows PhyParts analysis indicating the low support of bipartitions found in gene trees with dark red having support of 1.

**Table S1:** Names and characteristics of the genome assemblies used in this article.

| Organism | Name | Date | Assembly level |
| --- | --- | --- | --- |
| *Tyto furcata pratincola* (barn owl) BGI | ASM68720v1 | 05.08.2014 | Scaffold |
| *Taeniopygia guttata* (zebra finch) | Taeniopygia_guttata-3.2.4 | 02.08.2013 | Chromosome |
| *Ficedula albicollis* (collared flycatcher) | FicAlb1.5 | 06.27.2013 | Chromosome |
| *Gallus gallus* (chicken) | Gallus_gallus-5.0 | 12.16.2015 | Chromosome |

**Table S2**: The 57 Sanger sequenced genes used in this study with their names and accession numbers, number of exon (#), and start and stop codons positions.

| **Gene name** | **Accession number** | **Sequence length (bp)** | **# exon** | **Start codon position** | **Stop codon position** |
| --- | --- | --- | --- | --- | --- |
| AGRP | KU712271 | 664 | 3 | 78 | 539 |
| AR | KX108735 | 2504 | 8 | 3 | 2162 |
| ASIP | KU712317 | 1520 | 7 | 211 or 217 or 238 | 613 |
| CORIN | MK387693 | 3393 | 22 | 1 | 3393 |
| CPE | KX013118 | 1962 | 9 | ND | 1327 |
| CREB1 | KU712306 | 1191 | 8 | 214 | 1189 |
| CTHRC1 | MK387696 | 681 | 4 | ND | 681 |
| CTSL1 | KX013126 | 1112 | 7 | 102 | 1110 |
| CTSL2 | KX013127 | 1375 | 7 | 57 | 1071 |
| CYP11A1 | KX108742 | 1343 | 8 | 23 | 1342 |
| CYP19A1 | KX108750 | 1431 | 8 | 26 | 1429 |
| DCT | KU712274 | 1862 | 8 | 200 | 1766 |
| EEF1A | KU712278 | 1389 | 7 | 1 | 1387 |
| ESR1 | KX108738 | 2608 | 8 | 71 | 1835 |
| ESR2 | KX108753 | 1665 | 8 | 1 | 1663 |
| ESRRB | KX108747 | 1544 | 6 | 14 | 1313 |
| FAM20C | MK387697 | 1689 | 12 | 76 | 1689 |
| FURIN | KX013115 | 3297 | 15 | 124 | 2491 |
| GAPDH | KU712279 | 1054 | 10 | ND | 1052 |
| GR_NR3C1 | KX108749 | 2380 | 8 | 1 | 2320 |
| HPRT | KU712280 | 1106 | 8 | ND | 698 |
| HSD17B1 | KY433287 | 1141 | 5 | 85 | ND |
| HSD17B3 | KX108741 | 846 | 8 | 1 | 844 |
| KIT receptor | KU712281 | 2907 | 21 | 22 | 2905 |
| MBTPS1 | KX013131 | 3183 | 23 | 1 | 3181 |
| MC1R | KR018388 | 1312 | 1 | 344 | 1288 |
| MC2R | KY189303 | 1032 | 1 | 73 | 1030 |
| MC3R | KY189305 | 1389 | 1 | 172 | 1015 |
| MC4R | KY189307 | 1537 | 1 | 529 | 1522 |
| MC5R | KY189310 | 1123 | 1 | 136 | 1111 |
| MITF-B | KU712285 | 1656 | 9 | 1 | 1558 |
| MLANA | KY433283 | 590 | 5 | 69 | 410 |
| MR_NR3C2 | KX108754 | 3108 | 8 | 112 | 3055 |
| MRAP2 | MK387698 | 624 | 3 | 1 | 624 |
| OCA2 | KU712287 | 2627 | 23 | 72 | 2625 |
| PAM | KX013120 | 3339 | 25 | 415 | 3339 |
| PCSK1 | KU712308 | 3614 | 14 | 25 | 2257 |
| PCSK2 | KU712310 | 3273 | 12 | 79 | 1990 |
| PCSK4 | KX013128 | 1820 | 12 | ND | 1798 |
| PCSK5B | KU712316 | 6992 | 37 | 376 | 5968 |
| PCSK6 | KX013129 | 2855 | 20 | ND | 2724 |
| PCSK7 | KX013130 | 2353 | 15 | ND | 2266 |
| PGR | KX108758 | 2354 | 8 | 1 | 2351 |
| PMEL17 | KY433285 | 1977 | 10 | ND | ND |
| POMC | KU712269 | 1449 | 3 | 315 | 1109 |
| RPL13 | KU712291 | 642 | 5 | 7 | 640 |
| SGS5 | KX013125 | 1070 | 5 | 444 | 1068 |
| SLC45A2 | KU712292 | 2667 | 7 | 95 | 1733 |
| SLC7A11 | KU712297 | 2316 | 12 | 780 | 2289 |
| SOX10 | MK387699 | 3183 | 3 | 31 | 1422 |
| SRD5A1 | KY433289 | 933 | 5 | ND | 651 |
| SRD5A2 | KX108740 | 786 | 5 | 19 | 784 |
| SRD5A3 | KY433290 | 818 | 5 | ND | 804 |
| TBP | KY433286 | 1483 | 7 | 135 | 1043 |
| TUBB3 | MK387700 | 1353 | 4 | 1 | 1353 |
| TYR | KU712303 | 1941 | 5 | 91 | 1678 |
| TYRP1 | KU712300 | 1858 | 7 | 248 | 1858 |
| ND means not determined | |  |  |  |  |

**Table S3: (A)** List of the lost proteins in the avian genome of Table S3 of Warren (Warren et al., 2017) that were partially retrieved in the European barn owl transcriptome. The percent of identity of the European barn owl transcripts to the proteins are noted with Asterix (* ≥ 60 %, **≥ 80 %, *** =100 %). (**B**) List of the lost chicken proteins of Table S4 of Warren (Warren et al., 2017) that were partially or completely retrieved in the European barn owl transcriptome. Only BSCL2, CCDC155, DNAAF3, FLT3LG, HAUS4, IRGC, PRSS8, SAMD1 SERTAD1, SWSAP1, TGFBR3L, THTPA, TSR2 were not found in the European barn owl. The percent of identity of the European barn owl transcripts to the proteins are noted with Asterix (* ≥ 60 %, **≥ 80 %, *** ≥ 95 %).

**A**

| **SwissProt Name** | **Transcript**  **Name** |
| --- | --- |
| Q8WXF3 | c176876_g2_i1 |
| Q9BYM8 | c180396_g6_i3 |
| Q6UW68 | c259091_g1_i1 |
| Q9NRA0* | c179474_g7_i2 |
| Q6VY07* | c171186_g2_i1 |
| Q9UMN6* | c178299_g8_i5 |
| P08247* | c164876_g1_i2 |
| Q15904* | c164474_g1_i1 |
| Q6PCT2* | c180120_g3_i1 |
| O43448 | c180956_g3_i7 |
| P30518* | c210526_g1_i1 |
| Q9NS66* | c131196_g1_i1 |
| P48029* | c180975_g3_i1 |
| Q9UHI5** | c179955_g5_i7 |
| Q4KMP7** | c178168_g2_i1 |
| P49840** | c175275_g7_i1 |
| O75145** | c167286_g2_i2 |
| P17612** | c179322_g8_i5 |
| Q86U42*** | c40492_g1_i1 |

**B**

| **Protein Names** | **Barn owl transcripts** |
| --- | --- |
| ABCF1** | c180223_g2_i1 |
| ADCK5* | c178305_g21_i5 |
| ALDH16A1 | ND |
| ALKBH7* | c136475_g1_i1 |
| ARHGAP9 | c182122_g1_i8 |
| ATF6B* | c168487_g1_i5 |
| ATG9B | ND |
| ATP2B3** | c177154_g10_i1 |
| B4GALNT1* | c172181_g11_i2 |
| B9D2* | c170359_g1_i2 |
| BAG6* | c182584_g12_i2 |
| BAX* | c182134_g12_i1 |
| BCAT2* | c179864_g1_i3 |
| BEST2* | c266638_g1_i1 |
| BOLA2* | c180885_g3_i4 |
| BSCL2 | ND |
| C18orf54 | c171390_g3_i8 |
| C19orf54 | c175658_g1_i2 |
| C2orf68 | c167141_g1_i2 |
| CALM3*** | c118581_g1_i1 |
| CARM1** | c175260_g1_i1 |
| CCAR2 | ND |
| CCDC106* | c153522_g1_i1 |
| CCDC115 | ND |
| CCDC155 | c177683_g1_i8 |
| CCDC22 | c172379_g1_i4 |
| CCDC68 | c182088_g5_i4 |
| CD22 | c178955_g1_i7 |
| CD2BP2* | c181900_g10_i1 |
| CD37 | c179644_g15_i9 |
| CDK4* | c164502_g1_i1 |
| CFP | c174117_g1_i1 |
| CLIC1 | ND |
| COL5A3* | c179977_g6_i1 |
| CTDSP2*** | c179742_g2_i1 |
| CYC1** | c158684_g1_i1 |
| DBP** | c179861_g4_i2 |
| DCAF15* | c179378_g17_i5 |
| DCC** | c180441_g28_i1 |
| DEDD2 | ND |
| DMPK** | c175028_g1_i4 |
| DMWD** | c173359_g2_i1 |
| DNAAF3 | ND |
| DTX3** | c178654_g12_i2 |
| EFNB3* | c166603_g2_i1 |
| EHMT2** | c171599_g3_i1 |
| ELAVL3** | c181783_g8_i3 |
| EMC10* | c167014_g5_i1 |
| EMC4 | c165566_g1_i3 |
| EMP3 | c178331_g1_i8 |
| EPOR | c176213_g1_i5 |
| ERF*** | c142958_g1_i1 |
| FAM3A** | c180589_g10_i6 |
| FAM57B* | c24525_g1_i1 |
| FFAR3 | c310764_g1_i1 |
| FITM1 | c80892_g1_i1 |
| FKBPL | ND |
| FLT3LG | ND |
| FOXH1 | ND |
| FOXP3* | c174016_g3_i1 |
| GIPC1** | c182550_g9_i1 |
| HAUS4 | ND |
| HIPK4* | c167947_g2_i1 |
| HIRIP3 | ND |
| HSPA12B* | c173229_g1_i3 |
| INO80E** | c147084_g1_i3 |
| IRF2BP1* | c158415_g1_i1 |
| IRGC | ND |
| KCNK6 | ND |
| LIN37* | c179477_g3_i1 |
| MAP3K10 | ND |
| MBD6 | ND |
| MECP2* | c157459_g2_i1 |
| METTL1* | c176775_g1_i7 |
| MRPS18B* | c179762_g6_i7 |
| NACC1** | c178547_g5_i3 |
| NDUFA3* | c71149_g1_i1 |
| NPHS1 | c181782_g7_i4 |
| NUMBL** | c175599_g1_i4 |
| PCP2 | ND |
| PDLIM2* | c173188_g2_i1 |
| PDZD4** | c180776_g14_i6 |
| PET100 | ND |
| PFKFB1** | c181530_g4_i5 |
| PKMYT1 | ND |
| PLCB3** | c178289_g8_i2 |
| PLPPR2* | c181676_g4_i1 |
| POLI | c179624_g10_i7 |
| PPP1R14A** | c154375_g1_i3 |
| PPP1R16A* | c179245_g2_i3 |
| PRR12* | c135125_g1_i1 |
| PRRC2A* | c181416_g17_i1 |
| PRRT1** | c174742_g12_i1 |
| PRSS8 | ND |
| PSPN | ND |
| PTPN18 | ND |
| RAB27B** | c159433_g4_i1 |
| RAB2B*** | c171740_g2_i2 |
| RASIP1 | c174402_g5_i1 |
| RFX1* | c179124_g13_i5 |
| RPL13A* | c119451_g1_i1 |
| RPL18* | c206505_g1_i1 |
| RXRB* | c182664_g4_i4 |
| S1PR5 | ND |
| SALL2* | c176630_g1_i1 |
| SAMD1 | ND |
| SDR39U1 * | c181483_g14_i8 |
| SERTAD1 | ND |
| SLC39A4 | ND |
| STARD6 | ND |
| STX4 | c180918_g14_i3 |
| SWSAP1 | ND |
| SYT3* | c170572_g1_i1 |
| TGFBR3L | ND |
| THTPA | ND |
| TMED1* | c176556_g5_i4 |
| TMEM147* | c163996_g3_i1 |
| TMUB2* | c165580_g1_i3 |
| TOX4* | c179229_g1_i1 |
| TRMT1* | c182705_g2_i11 |
| TSPAN31* | c155983_g1_i1 |
| TSR2 | ND |
| TSSK4 | ND |
| TTC5 | c131875_g1_i1 |
| U2AF2 | ND |
| WNK3** | c172099_g2_i2 |
| WRAP53 | c180420_g7_i2 |
| YIPF2 | c168661_g1_i2 |
| ZNF574* | c181983_g5_i1 |

ND means not detected.

**Table S4**. Summary of the number of the paired-end reads obtained with the five RNAseq libraries.

| Tissue | # reads (bp) |
| --- | --- |
| Thalamus | 33'195'564 |
| Feather | 32'734'829 |
| Testis | 34'690'308 |
| Liver | 32'174'964 |
| Heart | 29'518'881 |
| Kidney | 30'645'603 |
| in total: | 192'960'149 |

# means number of.

**Table S5**: Selected species and their annotations used to build the phylogenetic trees.

| **Species code** | Ta**xID** | **Common name** | **Source** | **Release** |
| --- | --- | --- | --- | --- |
| ANAPL | 8839 | *Anas platyrhynchos* | OMA | Ensembl 73; BGI_duck_1.0; 24-AUG-2013 |
| ANOCA | 28377 | *Anolis carolinensis* | OMA | Ensembl 70; AnoCar2.0; 11-DEC-2012 |
| CHICK | 9031 | *Gallus gallus* | OMA | Ensembl 75; Galgal4; 7-FEB-2014 |
| FICAL | 59894 | *Ficedula albicollis* | OMA | Ensembl 73; FicAlb_1.4; 23-AUG-2013 |
| HUMAN | 9606 | *Homo sapiens* | OMA | Ensembl 80; GRCh38; 16-MAY-2015 |
| MELGA | 9103 | *Meleagris gallopavo* | OMA | Ensembl 70; UMD2; 11-DEC-2012 |
| MOUSE | 10090 | *Mus musculus* | OMA | Ensembl 86; GRCm38; 13-SEP-2016 |
| TAEGU | 59729 | *Taeniopygia guttata* | OMA | Ensembl 70; taeGut3.2.4; 12-DEC-2012 |
| XENTR | 8364 | *Xenopus tropicalis* | OMA | Ensembl 73; JGI_4.2; 23-AUG-2013 |
| Tyto alba BGI | 56313 | *Tyto furcata pratincola* (previously called *Tyto alba pratincola)* | BGI | Jarvis; Barn owl -O2 |
| Cathartes_aura | 43455 | *Cathartes aura* | BGI | Jarvis; Turkey vulture - CR38 |
| Colius_striatus | 57412 | *Colius striatus* | BGI | Gilbert; Speckled mousebird -TG2 |
| Haliaeetus_albicilla | 8969 | *Haliaeetus albicilla* | BGI | Gilbert; White tailed eagle -137926 |
| Haliaeetus_leucocephalus | 52644 | *Haliaeetus leucocephalus* | BGI | Jarvis; Bald Eagle |
| Leptosomus_discolor | 188344 | *Leptosomus discolor* | BGI | Gilbert; Cuckoo roller -CFM2 |
| Picoides_pubescens | 118200 | *Picoides pubescens* | BGI | Jarvis; Downy Woodpecker-B-21955, B-21955 |
| Tyto alba NCBI | 56313 | *Tyto furcata pratincola* (previously called *Tyto alba pratincola)* | NCBI | NCBI Tyto alba Annotation Release 100 |
| Strix occidentalis caurina | 311401 | *Strix occidentalis caurina* | Zenodo dataset | Hanna, Zachary R. *et al* (2017). Supplemental dataset for Northern Spotted Owl (*Strix occidentalis caurina*) genome assembly version 1.0 [Data set]. Zenodo. |
| Bubo blakistoni | 154486 | *Bubo blakistoni* | NCBI genome  Augustus annotations | NCBI Assembly Bubo blakistoni_ver1.0  Parameters: species=chicken |
| Athene cunicularia | 194338 | *Athene cunicularia* | NCBI | NCBI athCun1 Annotations |
