# Supplementary figures and images for: New genome assembly of the barn owl (Tyto alba alba)
Source: Ecol Evol. 2020 Feb 19;10(5):2284–98. doi: 10.1002/ece3.5991 (PMC7069322; doi:10.1002/ece3.5991)

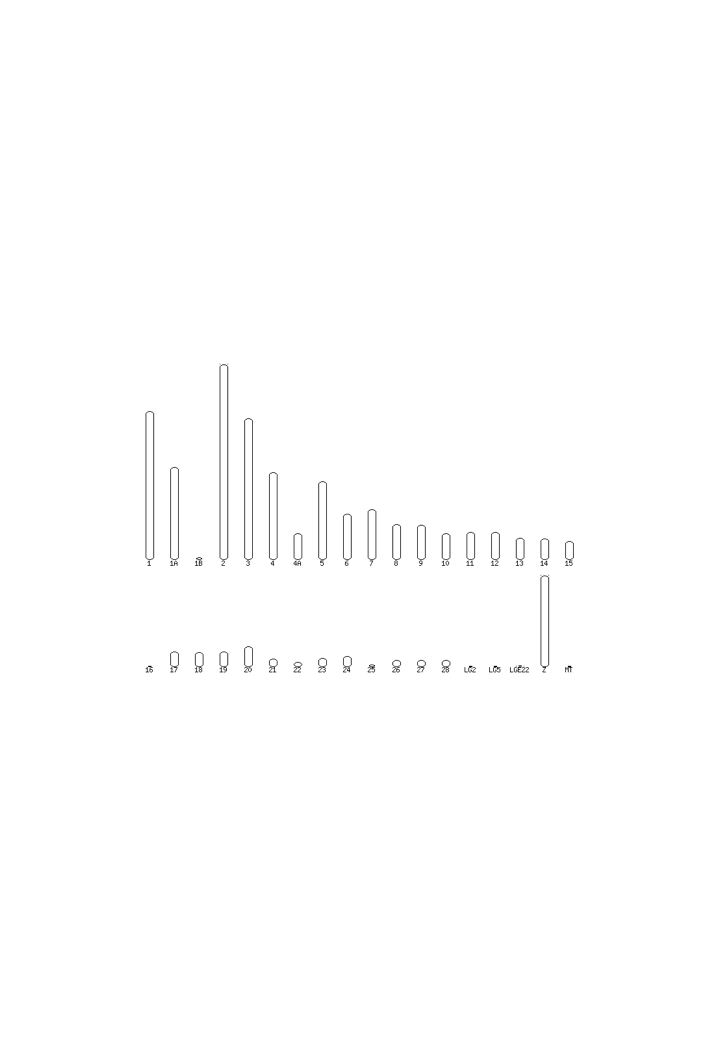

Supplement: Supplementary file 2 [file ECE3-10-2284-s002.tiff]

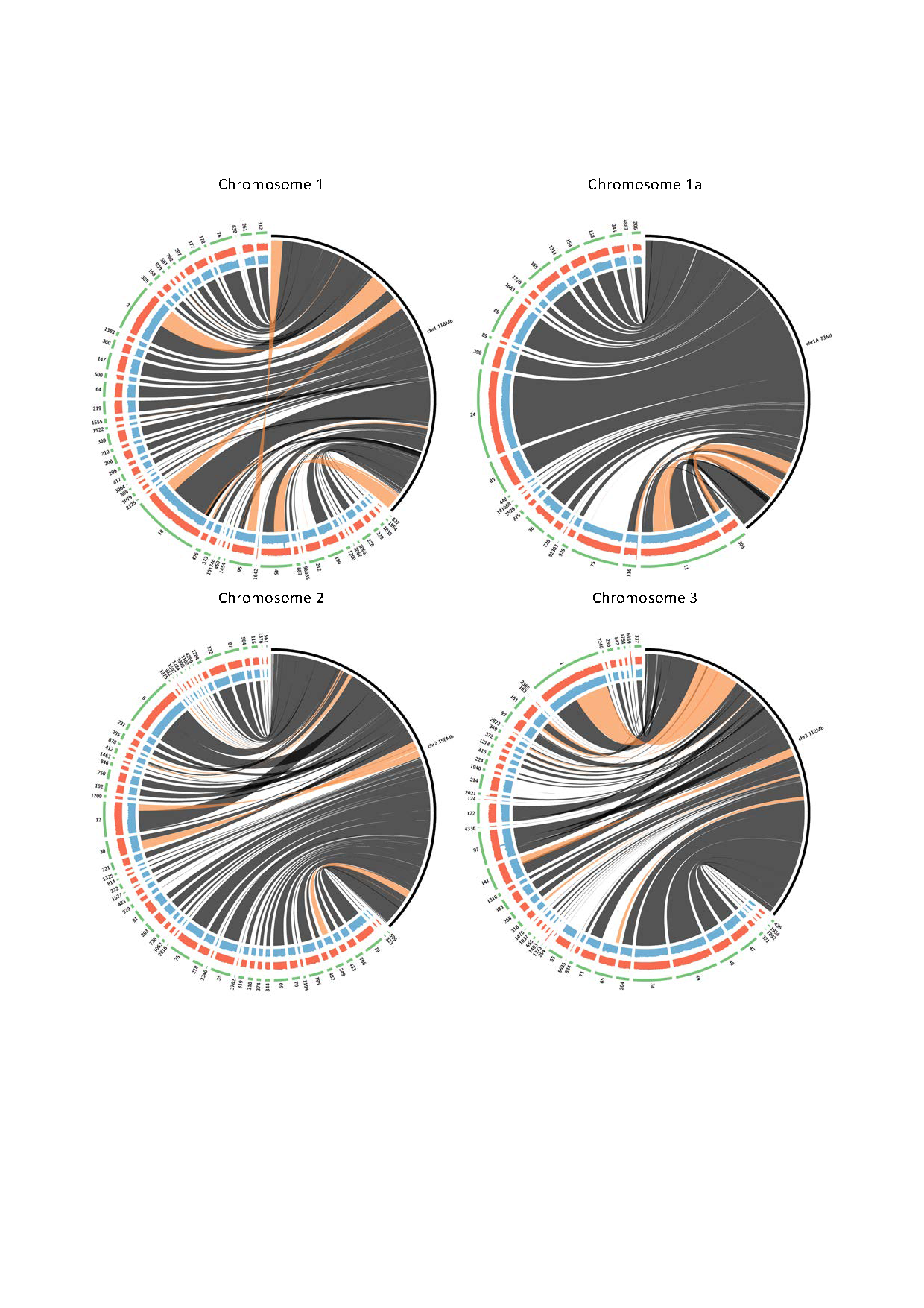

Supplement: Supplementary file 3 [file ECE3-10-2284-s003.tiff]

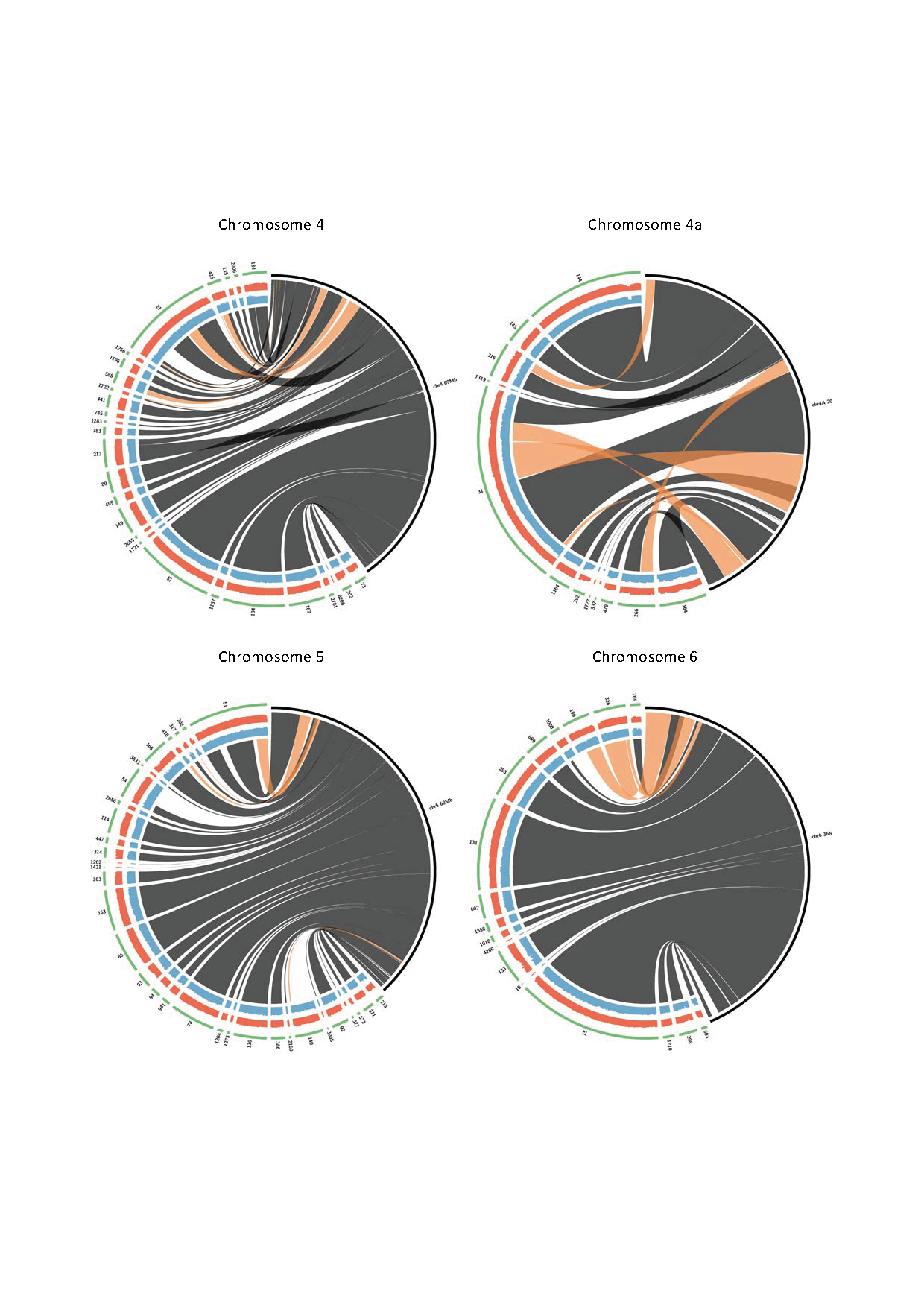

Supplement: Supplementary file 4 [file ECE3-10-2284-s004.tiff]

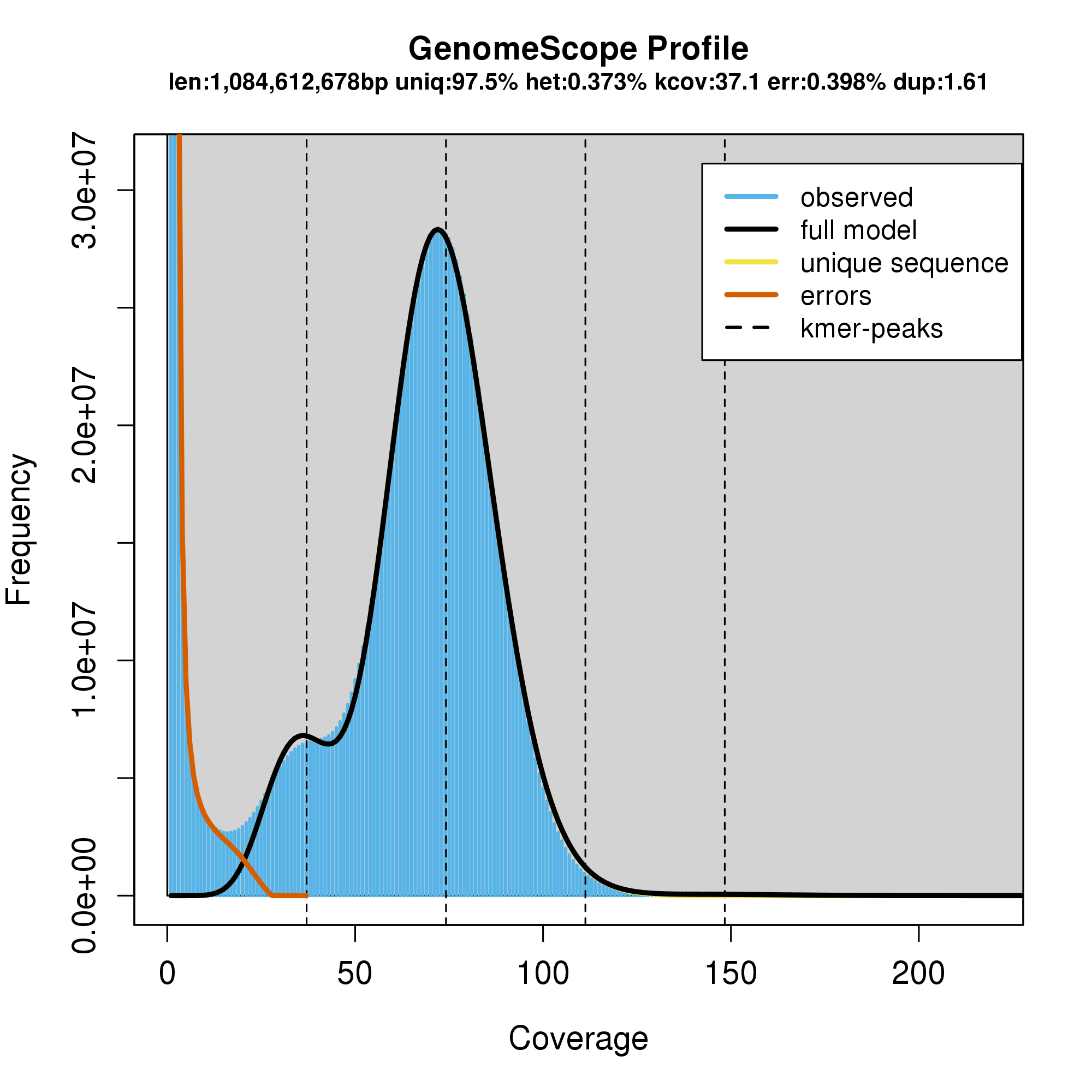

Supplement: Supplementary file 5 [file ECE3-10-2284-s005.jpg]
